# Crystal structure and mutational analysis of the human TRIM7 B30.2 domain provide insights into the molecular basis of its binding to glycogenin-1

**DOI:** 10.1016/j.jbc.2021.100772

**Published:** 2021-05-11

**Authors:** Christian J. Muñoz Sosa, Federico M. Issoglio, María E. Carrizo

**Affiliations:** 1 Centro de Investigaciones en Química Biológica de Córdoba (CIQUIBIC) - CONICET and Departamento de Química Biológica Ranwel Caputto, Facultad de Ciencias Químicas, Universidad Nacional de Córdoba, Córdoba, Argentina; 2 Instituto de Tecnologia Química e Biológica António Xavier, Universidade Nova de Lisboa (ITQB NOVA), Oeiras, Portugal; 3 Instituto de Química Biológica de la Facultad de Ciencias Exactas y Naturales (IQUIBICEN) - CONICET and Departamento de Química Biológica, Facultad de Ciencias Exactas y Naturales, Universidad de Buenos Aires, Buenos Aires, Argentina

**Keywords:** B30.2, crystal structure, E3 ubiquitin ligase, glycogenin, molecular dynamics, PRY-SPRY, protein–protein interaction, site-directed mutagenesis, TRIM7, BTN3A1, Butyrophilin 3A1, CBD, chitin-binding domain, CC, coiled-coil, CD, circular dichroism, GN1, glycogenin-1, GNIP, Glycogen Interacting Protein, MD, Molecular Dynamics, PDB, Protein Data Bank, RING, Really Interesting New Gene, RMSD, Root Mean Square Deviation, SASA, solvent accessible surface area, SOCS, suppressor of cytokine signaling, TRIM, TRIpartite Motif

## Abstract

Tripartite motif (TRIM)7 is an E3 ubiquitin ligase that was first identified through its interaction with glycogenin-1 (GN1), the autoglucosyltransferase that initiates glycogen biosynthesis. A growing body of evidence indicates that TRIM7 plays an important role in cancer development, viral pathogenesis, and atherosclerosis and, thus, represents a potential therapeutic target. TRIM family proteins share a multidomain architecture with a conserved N-terminal TRIM and a variable C-terminal domain. Human TRIM7 contains the canonical TRIM motif and a B30.2 domain at the C terminus. To contribute to the understanding of the mechanism of action of TRIM7, we solved the X-ray crystal structure of its B30.2 domain (TRIM7^B30.2^) in two crystal forms at resolutions of 1.6 Å and 1.8 Å. TRIM7^B30.2^ exhibits the typical B30.2 domain fold, consisting of two antiparallel β-sheets of seven and six strands, arranged as a distorted β-sandwich. Furthermore, two long loops partially cover the concave face of the β-sandwich defined by the β-sheet of six strands, thus forming a positively charged cavity. We used sequence conservation and mutational analyses to provide evidence of a putative binding interface for GN1. These studies showed that Leu423, Ser499, and Cys501 of TRIM7^B30.2^ and the C-terminal 33 amino acids of GN1 are critical for this binding interaction. Molecular dynamics simulations also revealed that hydrogen bond and hydrophobic interactions play a major role in the stability of a modeled TRIM7^B30.2^-GN1 C-terminal peptide complex. These data provide useful information that could be used to target this interaction for the development of potential therapeutic agents.

Tripartite motif (TRIM) proteins constitute a family of over 80 members encoded in the human genome (1) that participate in diverse cellular processes including proliferation, differentiation, apoptosis, autophagy, oncogenesis, innate immunity, and viral replication (reviewed in (1, 2, 3)). They share a multimodular architecture with a conserved N-terminal TRIM, also called RBCC motif, and a variable C-terminal region. The TRIM/RBCC motif includes a RING (Really Interesting New Gene) finger domain (R), one or two B-box domains (B), and a coiled-coil region (CC). The RING finger, which is present in most but not all TRIM proteins, is a cysteine-rich zinc-binding domain that confers E3 ubiquitin ligase activity (4). B-boxes, like the RING finger domain, are also zinc-binding motifs but with a less well-characterized function; they have been shown to regulate RING domain function, higher-order self-assembly, and substrate recognition (5, 6). The CC domain, on the other hand, is mainly involved in dimerization/oligomerization of TRIM proteins and in promoting the formation of high molecular weight complexes (7, 8). The C-terminal region can contain one or more domains that appear to mediate the recognition of E3 ubiquitin ligase substrates and other targets (reviewed in (9)). The most common of these C-terminal domains is the B30.2, also referred to as the PRY-SPRY domain, which is present in at least 30 human TRIM family members as well as in other unrelated proteins (10, 11).

Human TRIM7 is a 511-amino acid protein that displays the canonical TRIM/RBCC motif followed by a B30.2 C-terminal domain. It was first identified in a yeast two-hybrid screen for proteins that interact with glycogenin-1 (GN1), the autoglucosyltransferase responsible for the initiation of glycogen biosynthesis, and was therefore named GNIP1 (glycogen interacting protein isoform 1 (12)). The TRIM7/GNIP1–glycogenin interaction was shown to be mediated by the B30.2 domain, whereas the CC domain was involved in the protein self-association (13). These studies also demonstrated that GNIP2, a spliced isoform of TRIM7/GNIP1 containing the B30.2 domain and a segment of the CC region, stimulates GN1 autoglucosylation *in vitro* almost 4-fold (12). TRIM7 has also been shown to be an E3 ubiquitin ligase that plays a key role in the c-Jun/AP-1 activation pathway *via* Ras, by mediating the ubiquitination of the c-Jun co-activator RACO-1, thus leading to its stabilization (14). Consistent with these findings, TRIM7 depletion reduces c-Jun transcriptional activity, and its knockdown decreases the growth of xenograft lung tumors driven by Ras (14). Moreover, increased TRIM7 expression levels that correlate with tumor size have been described in hepatocellular carcinoma patients, an effect that is related to the regulation of the p38 MAP-kinase pathway (15). However, new evidences also describe TRIM7 as a suppressor of hepatocellular carcinoma progression *via* inhibition of the Src-mTORC1-S6K1 axis (16). TRIM7 is also involved in osteosarcoma metastasis and chemoresistance regulation (17). Through the activation of c-Jun/AP-1 signaling pathway, TRIM7 also promotes proliferation and migration of atherosclerotic vascular smooth muscle cells (18). Moreover, TRIM7 exhibits *in vitro* autoubiquitination activity, which could be attributed to its RING finger/B-box domains, and promotes glycogen accumulation in skeletal muscle when overexpressed (19). The relationship, if any, between the glycogenic effect of TRIM7 and its potential role as a GN1 activator is not known. Recent studies have also revealed that TRIM7 ubiquitinates Zika virus envelope protein, thereby favoring the entry of the virus into host cells and pathogenesis (20). Moreover, TRIM7 has been shown to inhibit norovirus infection in human cells (21) and the innate immune response against DNA virus through mediator of IRF3 activation ubiquitination and proteasome-mediated degradation (22). In addition, TRIM7 has an activating effect on the immune response mediated by Toll-like receptor 4 in macrophages (23).

As these evidences suggest, TRIM7 is emerging as a promising therapeutic target. In this context, its B30.2 domain may be particularly important because specificity determinants of many TRIM proteins lie in this domain (11). Thus, to contribute to the understanding of the mechanisms of action of TRIM7, we report here the X-ray crystal structure of its B30.2 domain, which constitutes the first structural study on this protein. We also define a putative binding interface in the TRIM7 B30.2 domain through the analysis of sequence conservation and assess the effect of different mutations on its interaction with GN1, the first protein identified as capable of interacting with this domain. The stability and dynamics of a docked complex between the TRIM7 B30.2 domain and the modeled structure of GN1 C-terminal peptide was further investigated using molecular dynamics (MD) simulations.

## Results

### Overall structure of the TRIM7 B30.2 domain

**Figure 1:**
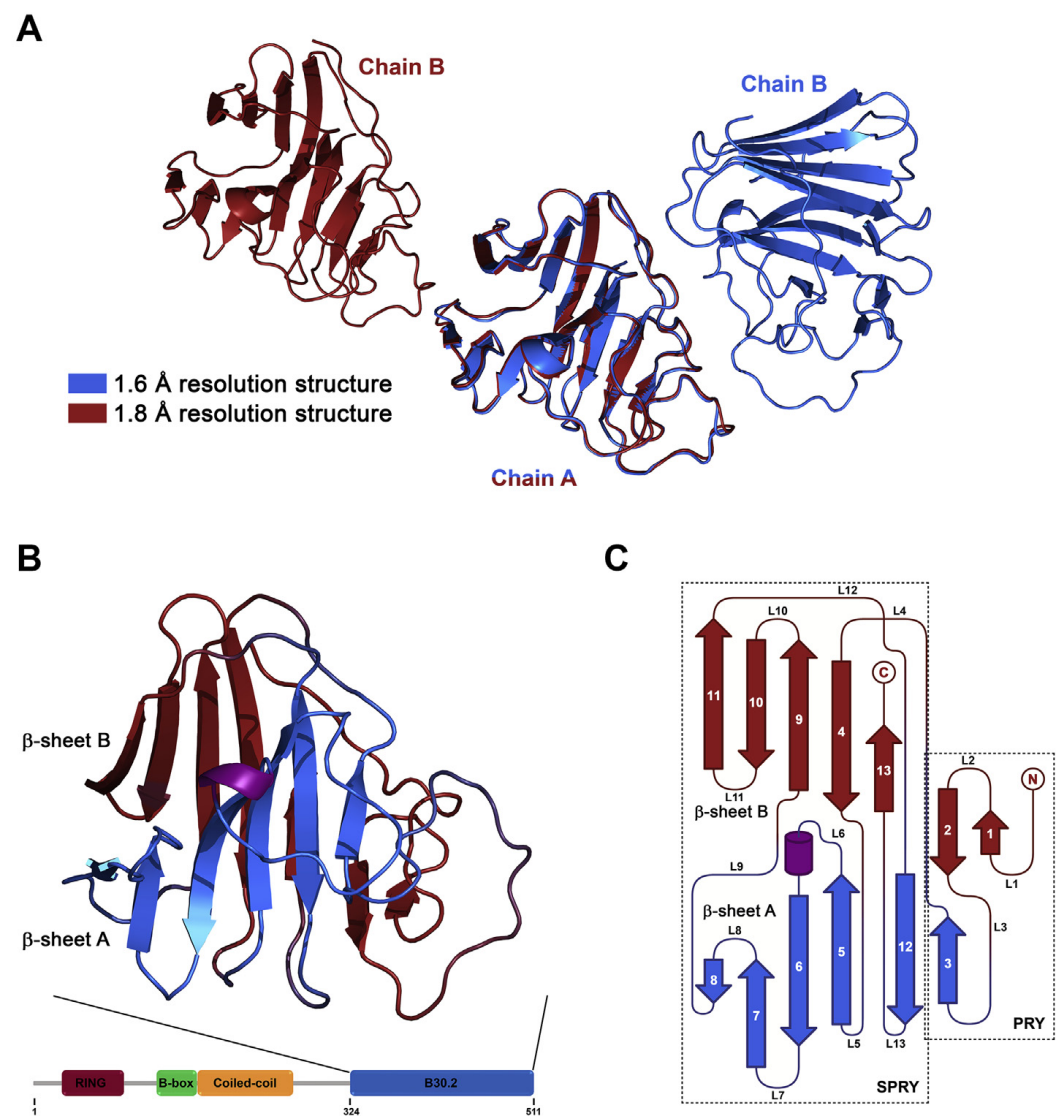
**Overall structure of TRIM7**^**B30.2**^**.***A*, superimposition of the asymmetric unit of the 1.6 Å (*blue*) and 1.8 Å (*red*) resolution structures. When superimposing the A chains of the two structures, the different arrangement of the B chains is made evident. *B*, ribbon diagram of TRIM7^B30.2^ showing β-sheet A and β-sheet B in *blue* and *red*, respectively. Secondary structure was assigned using DSSP (62). At the bottom of the figure, the domain architecture representation of TRIM7 highlights the position of the B30.2 domain. *C*, topology diagram of TRIM7^B30.2^ with the secondary structural elements colored as in *B*. PRY and SPRY subdomains are boxed in *dashed lines*. TRIM, tripartite motif.

The structure of the human TRIM7 B30.2 domain (named as TRIM7^B30.2^ from now on; the same convention will be used in all other B30.2 domains) has been solved in two different crystal forms (Table 1) using the molecular replacement method with the structure of the Butyrophilin 3A1 (BTN3A1) B30.2 domain (Protein Data Bank [PDB] ID 4N7I (24)) as a search model. Both crystal forms contain two molecules in the asymmetric unit (Fig. 1*A*). Even though the packing of the molecules in the two crystal forms is different, the structure of the four independent molecules is very similar, showing backbone root mean square deviations (RMSD) smaller than 0.5 Å for all pairwise comparisons. The structure of chain A at the highest resolution (1.6 Å; PDB ID 6UMA) was used for the general description of TRIM7^B30.2^ and subsequent analysis.

**Table 1:** Data collection and refinement statistics

	Crystal grown by hanging drop method (PDB ID 6UMA)	Crystal grown by sitting drop method (PDB ID 6UMB)
Data collection		
Space group	P2_1_2_1_2_1_	P2_1_2_1_2_1_
a (Å)	41.94	62.36
b (Å)	62.07	67.44
c (Å)	128.76	85.69
Resolution range (Å)	20.69–1.60 (1.63–1.60)	50.42–1.80 (1.84–1.80)
Observed reflections	237,684	193,627
Independent reflections	44,099	33,860
R_merge_ (%)^a^	4.2 (33.7)	7.6 (54.6)
I/σ (I)	11.3 (1.7)	10.7 (2.9)
Completeness (%)	97.6 (90.5)	99.6 (97.8)
Multiplicity	5.4 (5.3)	5.7 (5.9)
CC_1/2_	0.999 (0.959)	0.995 (0.917)
Wilson B-factor	20.9	18.5
Refinement		
Reflections in refinement	41,804	32,144
R_cryst_ (%)^b^	17.40	18.41
R_free_ (%) (test set 5%)^c^	20.59	22.34
No. of atoms		
Protein	2824	2784
Ligand	40	22
Solvent	202	165
Protein molecules/ASU^d^	2	2
Average B-factor		
Protein	27.75	26.37
Ligand	40.45	27.78
Solvent	34.37	30.18
RMSD from ideal values		
Bond lengths (Å)	0.012	0.011
Bond angles (°)	1.793	1.685
Ramachandran Plot (%)		
Favored	97	96
Allowed	3	4
Outliers	0	0

The values in parentheses refer to the highest resolution shells.

a Rmerge = Σ*h*Σ*i* | I*ih* – <I*h*> |/Σ*h*Σ*i* <I*h*> where <I*h*> is the mean intensity of the *i* observations of reflection *h*.

b Rcryst = Σ | |Fobs| - |Fcalc| |/Σ |Fobs| where |Fobs| and |Fcalc| are the observed and calculated structure factor amplitudes, respectively. Summation includes all reflections used in the refinement.

c Rfree = Σ | |Fobs| - |Fcalc| |/Σ |Fobs| evaluated for a randomly chosen subset of 5% of the diffraction data not included in the refinement.

d Protein molecules per asymmetric unit.

TRIM7^B30.2^ structure reveals the typical B30.2 domain fold consisting of two antiparallel β-sheets of seven (β1, β2, β4, β9, β10, β11, and β13) and six (β3, β5, β6, β7, β8, and β12) strands, arranged as a distorted β-sandwich (Fig. 1, *B* and *C*). In addition, there is a short 3_10_ helix (residues 428–430) located immediately before β6 strand. N- and C-terminal ends are close to each other in the seven-stranded β-sheet. This β-sheet (β-sheet B) forms a slightly convex surface at one side of the β-sandwich, whereas the six-stranded β-sheet (β-sheet A) forms a concave surface on the other side, which is partly covered by the two long loops that connect β2–β3 strands (loop 3) and β5 strand-3_10_ helix (loop 6). The high structural similarity between the four molecules from the two crystal forms and the relatively low B-factor values for the residues of these loops suggest that their conformation is quite stable, most likely because of interactions (hydrogen bonds and to a lesser extent hydrophobic contacts) within and between one another and with residues from loops 1 and 12 and the β12 strand.

Sequence homology and evolutionary studies indicate that the B30.2 domain is a fusion of two subdomains, named PRY and SPRY, forming a single structural module. According to the Pfam database assignment, in TRIM7^B30.2^, the PRY domain encompasses residues 344 to 392 (β1-β3 strands), whereas the SPRY domain includes residues 396 to 509 (β4-β13 strands) (Fig. 1*C*).

### Structural comparison of TRIM7^B30.2^ with other B30.2 domains

**Figure 2:**
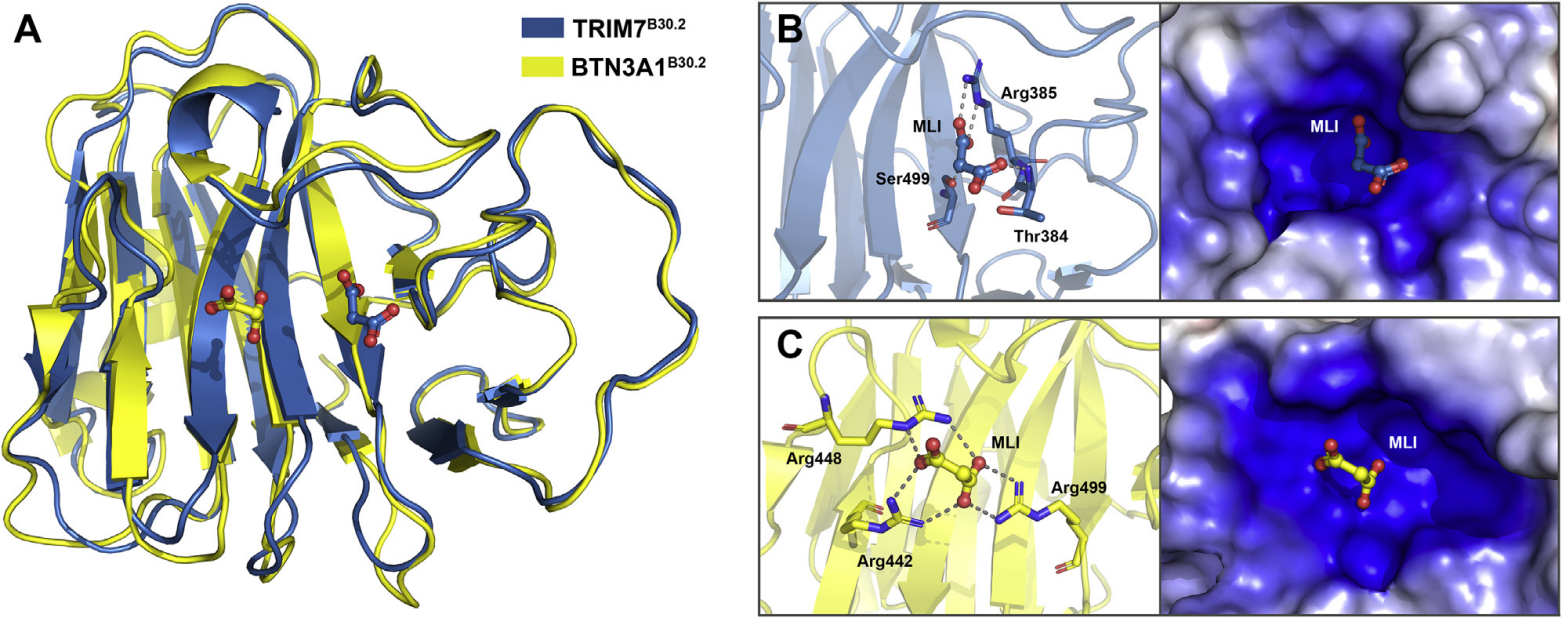
**Structural comparison of TRIM7 and BTN3A1 B30.2 domains and their binding to malonate**. *A*, backbone superimposition of TRIM7 (*blue*) and BTN3A1 (*yellow*, PDB code 5LYG) B30.2 domains with bound malonate (MLI). In both cases, malonate is shown in ball-and-stick representation. *B* and *C*, *left panels*, interactions of TRIM7 (*B*) and BTN3A1 (*C*) B30.2 domains with malonate. Hydrogen-bonding interactions are shown as dashed lines. *B* and *C*, *right panels*, electrostatic potential surface of malonate binding pockets of TRIM7 and BTN3A1 B30.2 domains calculated with contours from −5 kT/e (*red*) to +5 kT/e (*blue*) and positioned as in *left panels*. TRIM, tripartite motif; BTN3A1, butyrophilin 3A1.

A structural similarity search using the Dali server (25) revealed that the closest homolog to TRIM7^B30.2^ was BTN3A1^B30.2^ (PDB ID 4N7I, Z-score = 28.9, RMSD = 1.1 Å over 187 residues (24)), which curiously does not belong to the TRIM protein family (Fig. 2*A*). Among human proteins, BTN3A1^B30.2^ also exhibits a high sequence similarity with TRIM7^B30.2^ showing 41.3% identity. The other top matches from the Dali server analysis were the B30.2 domains of mouse TRIM21 (PDB ID 3ZO0, Z-score = 28.1, RMSD = 1.3 Å (26)), human TRIM25 (PDB ID 6FLM, Z-score = 27.8, RMSD = 1.5 Å (27)), human TRIM20/Pyrin (PDB ID 2WL1, Z-score = 26.0, RMSD = 1.5 Å (28)), human TRIM72 (PDB ID 3KB5, Z-score = 25.6, RMSD = 1.7 Å (29)), human TRIM14 (PDB ID 6JBM, Z-score = 25.7, RMSD = 1.6 Å (30)), and human TRIM65 (PDB ID 7JL4, Z-score = 23.7, RMSD = 1.9 Å (31)).

Despite its high structural and sequence similarity, unlike BTN3A1^B30.2^, which would be able to form dimers (24, 32), TRIM7^B30.2^ is monomeric both in solution on the basis of gel filtration chromatography (Fig. S1) and in the crystals, according to the quaternary assembly analysis with the PISA server (33). In fact, the asymmetric unit of both crystal forms includes two molecules but in a different spatial arrangement (Fig. 1*A*). The structure of the full-length intracellular domain of BTN3A1 exhibits two potential dimer interfaces (32), one of which involves only the B30.2 domain. A possible explanation for the different quaternary structure of TRIM7^B30.2^ and BTN3A1^B30.2^ arises from the analysis of the conservation of the amino acids involved in the formation of the dimer of the latter (Fig. S2). This analysis shows that the residues that would contribute to the interaction from one of the protomers of the BTN3A1^B30.2^ dimer and their equivalent in TRIM7^B30.2^ have an identity of 35.7% and a similarity of 42.9%, whereas those from the second protomer are much less conserved (identity 0%, similarity 28.6%). Among the latter are Trp350, Tyr352, and Trp391 (equivalent to Thr382, Thr384, and Leu423 from TRIM7) which would be particularly important for the interaction because they participate in stable contacts as revealed by MD simulations (32).

During the refinement process, additional electron densities connected to Cys378, Cys390, and Cys501 were observed in the F_obs_-F_calc_ map in both crystal forms (Fig. S3). As in the case of BTN3A1^B30.2^ (PDB ID 4N7I (24)), the extra electron density was attributed to the modification of the cysteines by the β-mercaptoethanol present in the crystallization medium, yielding S,S-(2-hydroxyethyl)thiocysteine. Only Cys455, whose side chain points into the core of the β-sandwich, appears not to be modified according to the electron density maps.

### Mapping of TRIM7^B30.2^ potential binding interfaces

**Figure 3:**
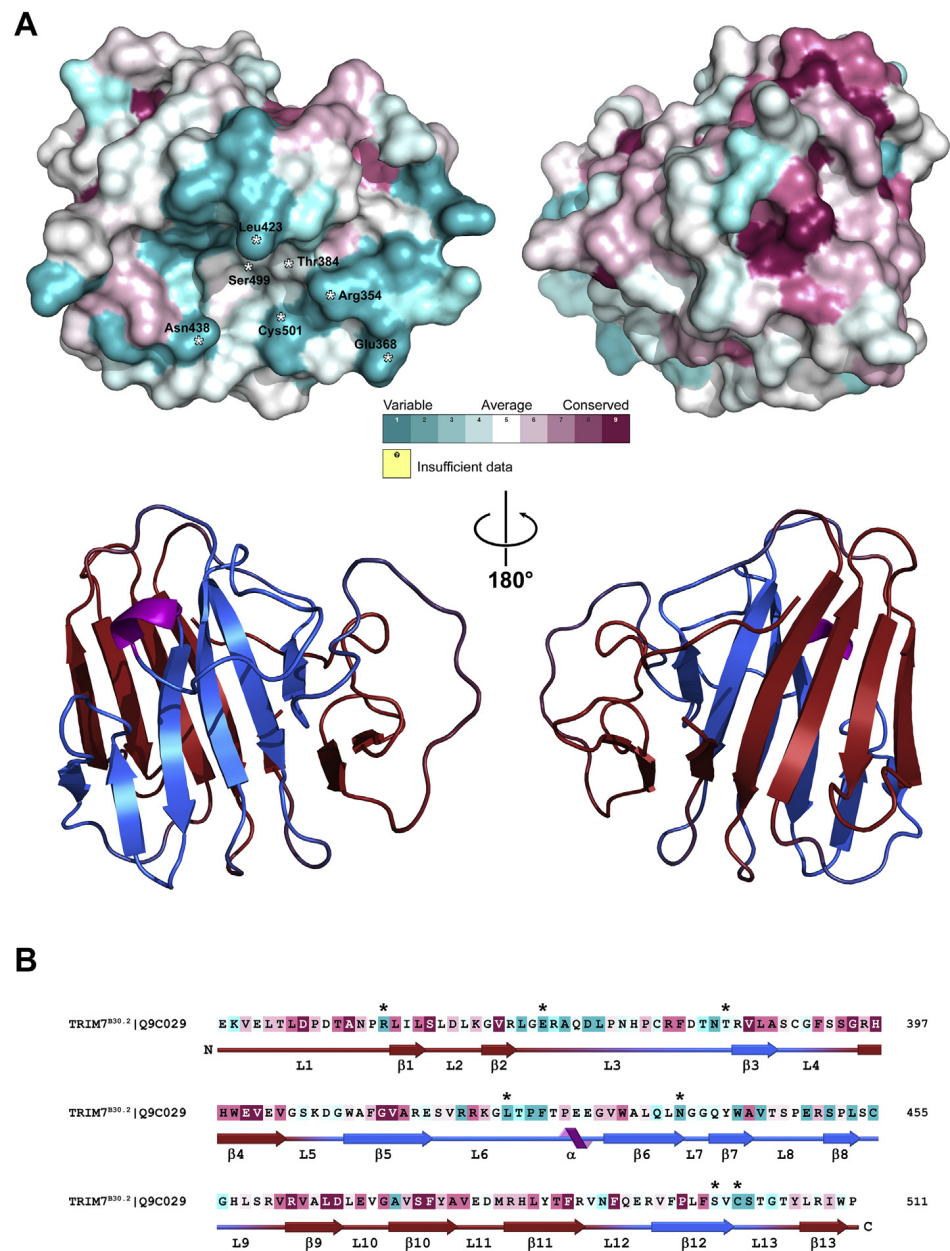
**Sequence conservation analysis of TRIM7**^**B30.2**^**homologs.***A*, mapping of conservation scores onto the molecular surface of TRIM7^B30.2^ (*top*). The color scheme was generated using the ConSurf server (37) based on an alignment of sequences from the B30.2 domain of 42 human proteins (Fig. S4). The amino acids are colored by their conservation degree according to the color-coding bar, from the most conserved (*dark purple*) to the most divergent (*dark cyan*). The structure is shown in two orientations rotated by 180 degrees about the indicated axis as in the cartoon representation (*bottom*). *B*, TRIM7^B30.2^ sequence colored according to the conservation color scale used in (*A*). The secondary structure elements for TRIM7^B30.2^ are indicated below the sequence. In both (*A*) and (*B*), *asterisks* indicate the amino acids that were mutated for the analysis of the interaction with glycogenin-1. TRIM, tripartite motif.

**Figure 4:**
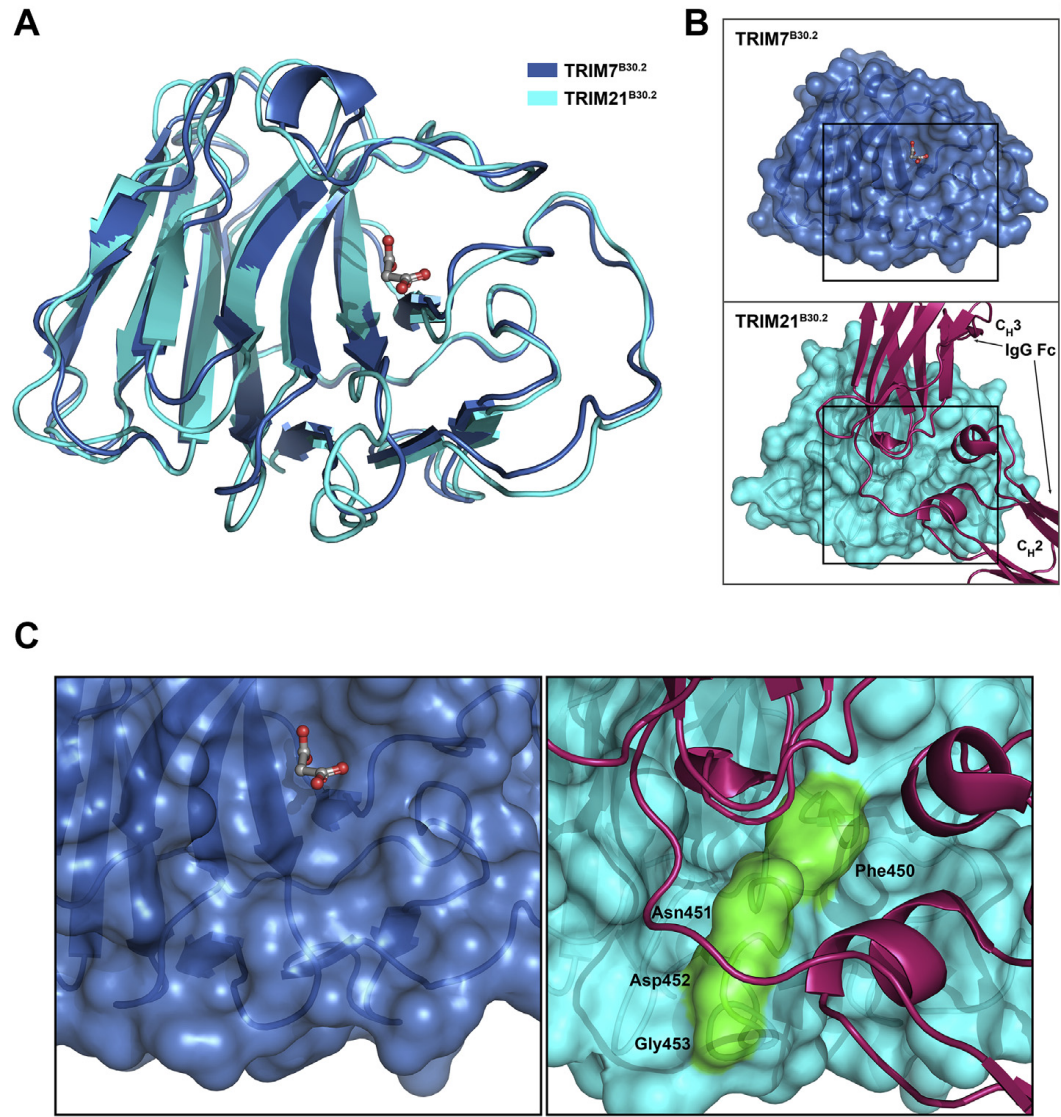
**Structural comparison of TRIM7 and TRIM21 B30.2 domains.***A*, backbone superimposition of TRIM7 (*blue*) and TRIM21 (*cyan*, PDB code 2IWG) B30.2 domains. *B*, molecular surface representation of both proteins oriented as in (*A*). In this model, TRIM21^B30.2^ is in complex with its target IgG Fc; thus, the IgG domains of Fc, C_H_2, and C_H_3, are also represented in the image. *C*, close-up views of the *boxed areas* in (*B*). The ridge that separates the two binding pockets in TRIM21^B30.2^ is colored *green* (*right panel*). TRIM, tripartite motif.

Because the main function of the B30.2 domains appears to be to mediate interactions with different targets, we sought to define the potential TRIM7^B30.2^ binding region by comparison with other B30.2 domain sequences and structures. Given the high structural homology between TRIM B30.2 domains, the conserved residues might be presumably important in determining the domains’ general conformation, whereas their different binding specificity would be related to differences in their sequences. In this respect, Song has described the existence of four regions in B30.2 domains that exhibit significant variations both in their length and in their amino acid composition (34, 35). Using a similar criterion, in an effort to identify potentially important residues for TRIM7^B30.2^ binding specificity, a multiple sequence alignment of 42 human B30.2 domain sequences (37 from TRIM family proteins and five from other proteins, including Butyrophilins) was performed using the program Clustal Omega (36). The results were used to calculate the degree of conservation of each amino acid using the ConSurf server (37) (Fig. S4). Conservation scores were mapped onto the three-dimensional protein structure by the server using a representative coloring scheme (Fig. 3, *A* and *B*). The analysis revealed that most of the conserved residues are clustered on one face of the β-sandwich comprising β-sheet B and loops 1, 2, 4, 10, and 11 (Fig. 3*A*). In contrast, the vast majority of the nonconserved residues map on the opposite side and are located on loops 3, 6, 7, 8, and 13 and the solvent-exposed region of β-sheet A. Invariant residues corresponding to β-strands 3, 5, 6, and 12 of β-sheet A are mostly hydrophobic, with their side chains buried at the interface between the β-sheets, and are covered by loop 6 and to a lesser extent by loop 3. Regions of high variability corresponding to six loops, most of them analogous to those found to be variable in TRIM7^B30.2^, have also been described as part of the binding interface in the structure of the TRIM21^B30.2^ in complex with IgG Fc (38). Moreover, the face of TRIM7^B30.2^ on which variable residues are clustered matches with the canonical binding interface proposed from the analysis of TRIM21^B30.2^ (Fig. 4, *A* and *B*). TRIM21 residues Asp355, Trp381, Trp383, and Phe450 are essential for IgG Fc binding (38). In TRIM7, they are replaced by amino acids with very different properties, *i.e.,* Ala410, Gln436, Asn438, and Ser502, respectively. One of the major structural differences between TRIM7^B30.2^ and TRIM21^B30.2^ lies in the loop equivalent to loop 13 in TRIM7^B30.2^ that includes most of the residues involved in the interaction with IgG Fc (Asn451, Asp452 and Gly453). This loop is longer in TRIM21 and in addition to the mentioned amino acids contains the bulky residue Phe450; consequently, it constitutes a ridge that separates the two pockets that bind each of the two IgG Fc domains (Fig. 4*C*, right panel). This ridge is not present in TRIM7, and therefore, only the pocket centered on the nonconserved face, which is shallower than that of TRIM21, can be observed (Fig. 4*C*, left panel). All these sequence and localized structural divergences between both B30.2 domains suggest that the conformation of the TRIM7^B30.2^ binding partner and the characteristics of the TRIM7^B30.2^/partner interaction must be quite different from those observed in the TRIM21^B30.2^/IgG Fc complex.

A more detailed analysis of the TRIM7^B30.2^ pocket revealed that it is defined by amino acids from loops 3, 6, 8, and 13 and β-strands 5, 6, and 7, most of them variable residues. During the refinement process, electron density that fitted well to a malonate ion from the crystallization solution was observed in a positively charged cavity found on one side of the pocket, in all four TRIM7^B30.2^ molecules from both crystal forms (Fig. S5). Malonate is in the same position in three of the four molecules (chain A from the 1.6 Å resolution structure and both chains in the 1.8 Å resolution structure) hydrogen-bonded to Thr384, Arg385 and Ser499 (Fig. 2, *A* and *B*). The most external carboxylate of malonate also forms a bidentate salt bridge with the guanidinium group of Arg487 from a symmetry-related protein molecule. In the fourth molecule, this carboxylate group does not interact with a symmetry-related molecule and consequently exhibits a different orientation. A malonate ion was also observed in a topologically equivalent pocket in BTN3A1^B30.2^ (PDB ID 5LYG (39)) but at approximately 6 Å from the position that it occupies in TRIM7^B30.2^, as determined after superimposition of the two structures. It is bound to three arginines (Arg442, Arg448, and Arg499) that constitute a highly positive patch on the pocket surface (Fig. 2, *A* and *C*), which also interacts with hydroxy-methyl-butyl-pyrophosphonate (cHDMAPP) a synthetic analog of the phosphoantigen hydroxy-methyl-butyl-pyrophosphate (HMBPP) (39). In the same interface, TRIM14^B30.2^ also exhibits a positive pocket that includes four basic residues (Arg354, Lys365, Arg379, and Arg430) which are crucial for the binding of acidic peptides (30). This pocket is shallower than that found in TRIM7^B30.2^ mainly because of the presence of Arg430 at the position equivalent to Ser499. The central role of this pocket in the binding of ligands to BTN3A1^B30.2^, TRIM21^B30.2^, and TRIM14^B30.2^ suggests that it would also be important for the interaction between TRIM7^B30.2^ and its binding partners. Furthermore, despite almost certainly being a nonnatural ligand of TRIM7^B30.2^, malonate binding reveals the existence of a potential binding site for negatively charged regions of such partners.

**Figure 5:**
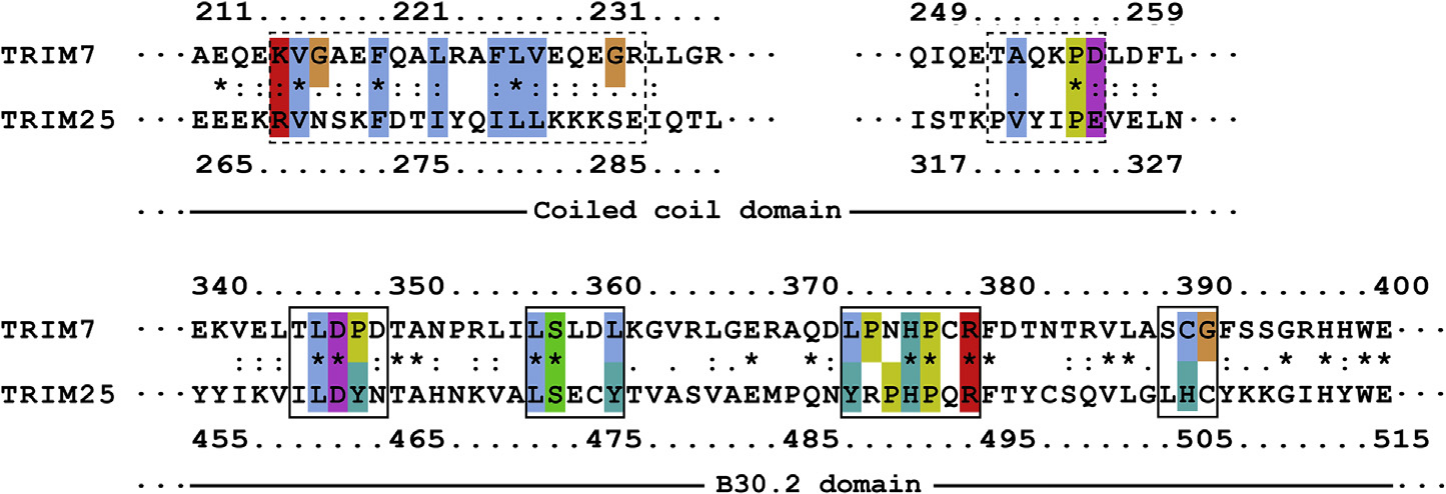
**Sequence alignment of TRIM7 and TRIM25 coiled-coil and B30.2 domains.** TRIM25 amino acids involved in the interaction between B30.2 and coiled-coil domains and their homologs in TRIM7 are boxed. TRIM7 and TRIM25 B30.2 domain residues are also structurally aligned (*solid line box*). Conserved amino acids are indicated by an *asterisk* (∗), strong amino acid group similarity by a *colon* (:), and weaker group similarity by a *single dot* (.). The color code indicates the different types of amino acids according to Clustal Omega (36). See Figure S6 for the complete sequence alignment. TRIM, tripartite motif.

A recent study describing the TRIM25 CC-B30.2 module crystal structure has shown that CC domains form dimers and bind the B30.2 domain (27). This binding involves TRIM25^B30.2^ regions spanning residues 460 to 464, 472 to 476, 488 to 494, and 504 to 506, which are equivalent to the regions corresponding to residues 345 to 349 (L1), 357 to 361 (L2), 373 to 379 (L3), and 389 to 391 (L4) of TRIM7^B30.2^, respectively. The first three regions have approximately 40% sequence identity between both proteins (Figs. 5 and S6) and show similar surface topology and electrostatic charge distribution (data not shown). The interaction also involves the regions that span the residues 269 to 287 and 320 to 326 from TRIM25-CC. According to a sequence alignment, these regions correspond to residues 215 to 233 and 273 to 279 of TRIM7 that have 15.8% identity/89.5% similarity and 16.7% identity/50.0% similarity, respectively, with TRIM25 sequences (Fig. 5 and S6). These observations suggest that the interaction between CC and the B30.2 domain could also occur in TRIM7.

### Determination of TRIM7 ^B30.2^ binding interface through mutational analysis

**Figure 6:**
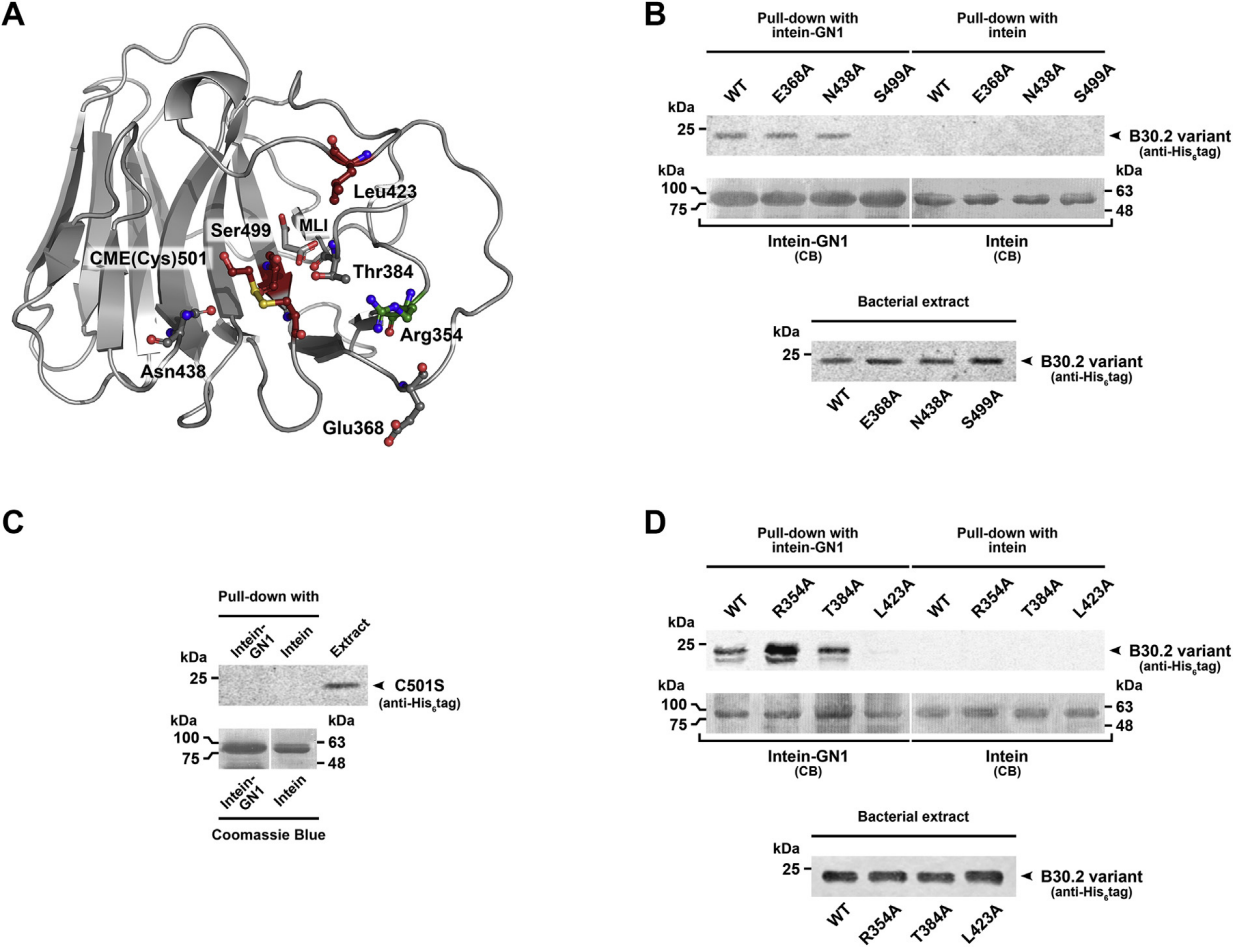
**Pull-down analysis of the binding between glycogenin-1 and TRIM7**^**B30.2**^**variants.***A*, ribbon representation of TRIM7^B30.2^ crystal structure with mutated amino acids (Arg354, Thr384, Glu368, Leu423, Asn438, Ser499, and CME(Cys)501) shown as *sticks*. *B*, chitin beads carrying intein-fused glycogenin-1 (GN1) were incubated with clarified extracts of *E. coli* overexpressing TRIM7^B30.2^ WT or the mutants Glu368Ala, Asn438Ala, and Ser499Ala according to Experimental procedures. TRIM7^B30.2^ variants bound to 10 μl of the chitin beads carrying GN1 were analyzed by immunoblot with an anti-His_6_ tag antibody. As a control, the same analysis was carried out using only intein. Blotted intein (molecular weight ≈ 55 kDa) and intein–GN1 (molecular weight ≈ 93 kDa) were identified after Coomassie Brilliant Blue (CB) staining of the membranes and used as loading control (*top*). Immunoblot analysis of 5 μl of the clarified extracts of *E. coli* overexpressing TRIM7^B30.2^ variants (*bottom*). 50 μl of these extracts were incubated with 20 μl of the chitin beads for the pull-down assays. *C*, same analysis as in (*B*) but using Cys501Ser mutant of TRIM7^B30.2^. *D*, same analysis as in (*B*) but with TRIM7^B30.2^ mutants Arg354Ala, Thr384Ala, and Leu423Ala. CME, S,S-(2-hydroxyethyl)thiocysteine; TRIM, tripartite motif.

To determine whether the binding site of TRIM7^B30.2^ is on the concave side of the β-sandwich as suggested by the analysis of its structure, we made the following mutations: Glu368Ala, Asn438Ala, and Ser499Ala (Fig. 6*A*). The first criterion for the choice of these residues was their variability according to the ConSurf server analysis described above (Figs. 3, *A* and *B* and S4). Furthermore, Glu368 and Ser499 are analogous to Asp488 and Trp621 of mouse TRIM25 (Glu483 and Trp616 in human TRIM25), respectively, whose mutation to Ala decreases the binding of TRIM25^B30.2^ to retinoic acid–inducible gene-I and its ubiquitination (40). Ser499 is also involved in the interaction with malonate (Fig. 2*B*). Asn438 is equivalent to Trp383 of human TRIM21, which is essential for IgG Fc binding (38). All mutants were analyzed by far-UV circular dichroism (CD) spectroscopy and have similar profiles to that of the WT (Fig. S7), suggesting that the mutations did not significantly modify the structure of TRIM7^B30.2^.

WT TRIM7^B30.2^ and the mutants were tested for binding to human GN1, the first known TRIM7^B30.2^-interacting protein (13). Pull-down assays were performed by incubating His_6_-tagged TRIM7^B30.2^ mutants with GN1 fused to chitin-binding domain (CBD)–intein and immobilized in chitin beads. As Figure 6*B* shows, TRIM7^B30.2^ Glu368Ala and Asn438Ala mutants binding to GN1 is similar to that of the WT domain. In contrast, the replacement of TRIM7^B30.2^ Ser499 by alanine abrogates the interaction with GN1. As a control, the clarified extracts of the bacteria overexpressing all the TRIM7^B30.2^ variants were also analyzed to confirm that they contained similar quantities of the proteins (Fig. 6*B*).

As the TRIM7^B30.2^ crystal structure showed, the β-mercaptoethanol included in the purification and crystallization to prevent precipitation at a high concentration, reacted with three of its four cysteines without significantly affecting the structure as revealed by CD analysis (Fig. S7). Given its proximity to Ser499, Cys501, one of the cysteines modified by β-mercaptoethanol, was mutated to serine and also used in GN1 binding assays. As shown in Figure 6*C*, mutant Cys501Ser exhibited a similar behavior to Ser499Ala, indicating that it is also likely involved in the interaction. These findings suggest that conversion of Cys501 to S,S-(2-hydroxyethyl)-thiocysteine by the β-mercaptoethanol derivatization could cause the loss of interaction with GN1, a detail to be taken into account when seeking to obtain co-crystals with a binding partner. Based on these results, additional TRIM7^B30.2^ single mutants were generated by replacing three amino acids located in the vicinity of Ser449 (*i.e.,* Arg354, Thr384, which is also involved in malonate binding, and Leu 423) with alanine. As in the case of the first mutants, they were analyzed by CD to verify that the mutations did not produce significant changes in the structure of TRIM7^B30.2^ (Fig. S7). These new mutants were also used in pull-down assays with GN1. As Figure 6*D* shows, Leu423Ala mutant does not significantly bind to GN1 while Arg354Ala mutant exhibits an approximately 2-fold increased binding affinity compared with TRIM7^B30.2^ WT. Instead, the substitution of Thr384 (Thr384Ala mutant) had no significant effect on the interaction with GN1. All these results not only indicate that Leu423, Ser499, and Cys501 participate in the interaction with GN1 but also confirm that the binding site is located in the concave face of TRIM7^B30.2^.

### Identification of the region of glycogenin-1 involved in the interaction with TRIM7

**Figure 7:**
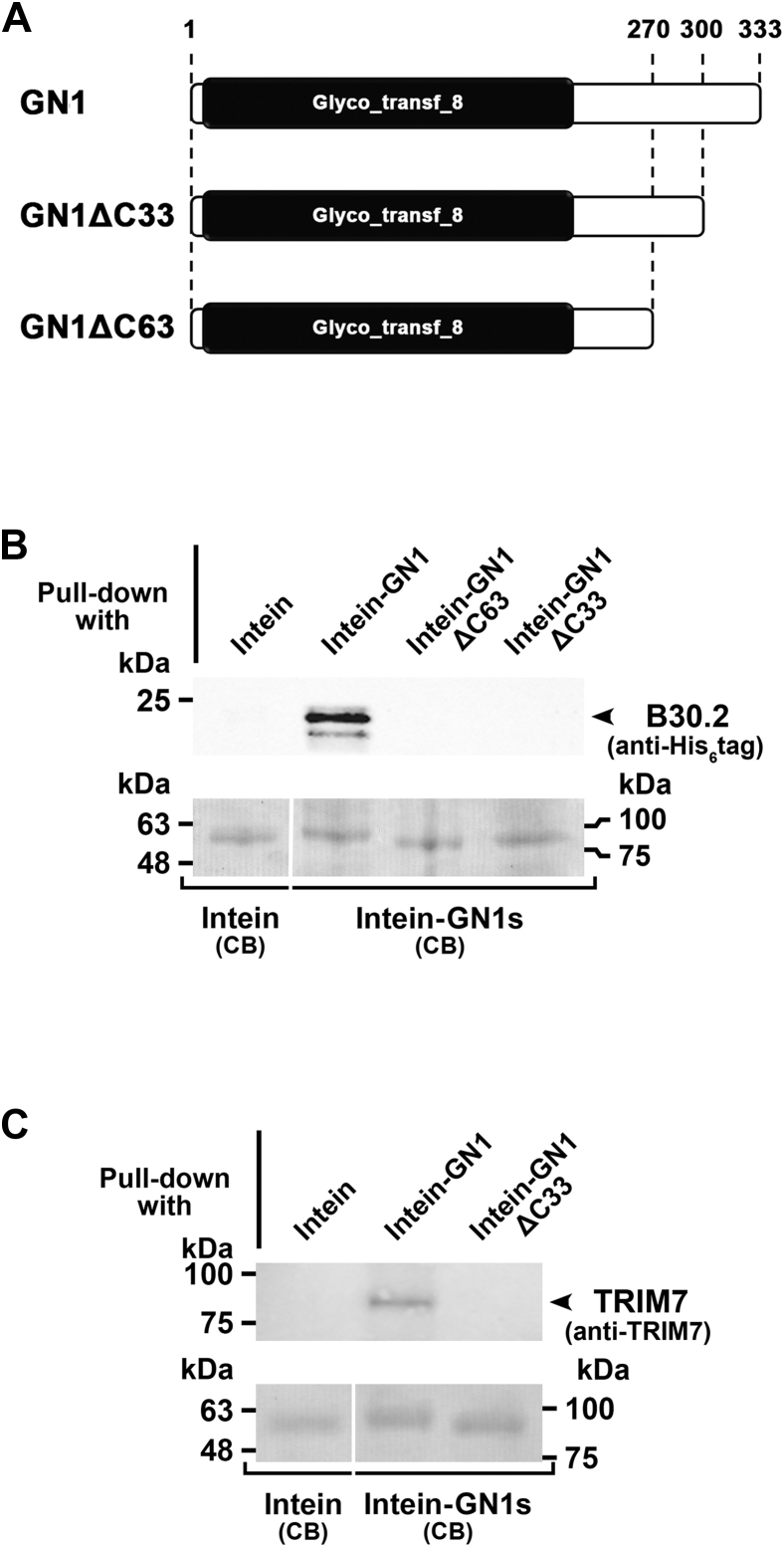
**Pull-down analysis of glycogenin-1 truncated mutants binding to TRIM7**^**B30.2**^**and TRIM7.***A*, schematic representation of the truncated mutants of glycogenin-1 used in the assays. *B*, chitin beads carrying intein-fused glycogenin-1 (GN1) or the truncated mutants GN1ΔC63 and GN1ΔC33 were incubated with clarified extracts of *E. coli* overexpressing TRIM7^B30.2^ WT and processed as in Figure 6*B*. Blotted intein (molecular weight ≈ 55 kDa), intein–GN1 (molecular weight ≈ 93 kDa), intein–GN1ΔC63 (molecular weight ≈ 86 kDa), and intein–GN1ΔC33 (molecular weight ≈ 89 kDa) were identified after Coomassie Brilliant Blue (CB) staining of the membranes and used as loading control. *C*, same analysis as in (*B*) but using TRIM7. In this case, an anti-TRIM7 antibody was used in the immunoblot. TRIM, tripartite motif.

To define the region of GN1 responsible for its interaction with TRIM7^B30.2^, C-terminal truncated versions of the enzyme lacking the last 63 (GN1ΔC63) and 33 amino acids (GN1ΔC33) (Fig. 7*A*) and fused to CBD–intein were tested for their ability to bind TRIM7^B30.2^ in pull-down experiments. As shown in Figure 7*B*, the deletion of the last 33 amino acids of GN1 results in the loss of interaction. More importantly, the same result was obtained when pull-down assays were performed using full-length TRIM7 (Fig. 7*C*). These results indicate that the region of interaction of GN1 with TRIM7 lies within residues 301 to 333.

### Molecular dynamics simulation analysis of the complex between TRIM7^B30.2^ and GN1-Cter

**Figure 8:**
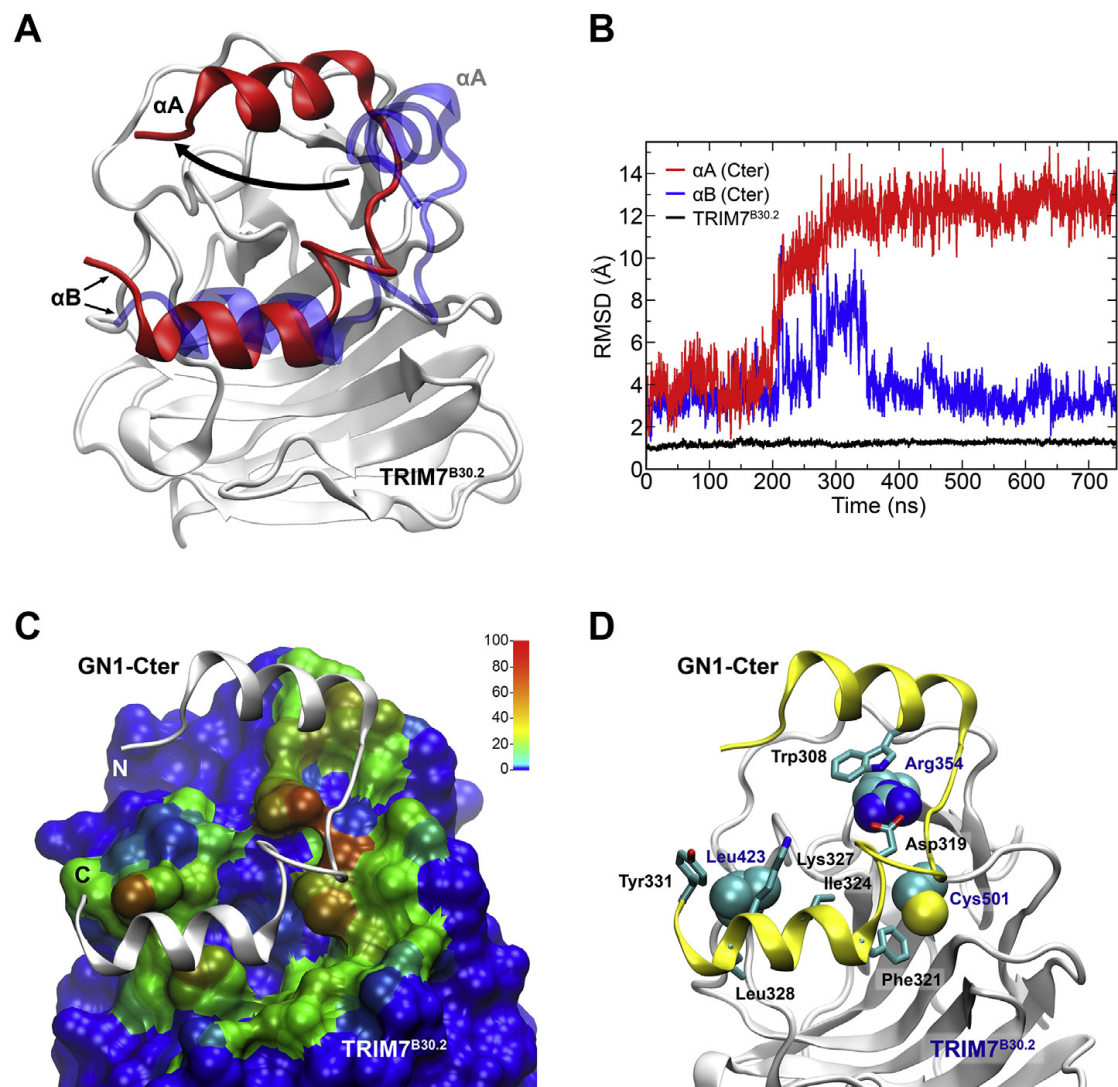
**Molecular dynamics simulation of the docked complex of TRIM7**^**B30.2**^**and GN1-Cter.***A*, modeled structure of the complex between TRIM7^B30.2^ (*white*) and GN1-Cter before (*transparent blue*) and after MD simulations (*red*). The arrow indicates the conformational rearrangement of GN1-Cter α-helix A (αA) during the simulation. *B*, root mean square deviation relative to the optimized structure for backbone atoms of TRIM7^B30.2^ (*black line*) and GN1-Cter α-helix A (*red line*), and α-helix B (*blue line*). C, the results of a contact map calculated for every residue in TRIM7^B30.2^ after convergence of the complex conformation were normalized and illustrated by color on a surface representation of the structure. GN1-Cter is represented as *white ribbons*. Color scale bar represents the percentage of simulation time in which TRIM7^B30.2^ residues are in close contact with those of GN1-Cter. D, TRIM7^B30.2^ residues with the highest values in the contact map analysis are represented as *spheres* while the GN1-Cter amino acids involved in the main interactions are depicted as *sticks*. TRIM, tripartite motif.

To get insights into the stability of the complex between TRIM7^B30.2^ and GN1, we combined protein–protein docking and all-atom MD simulations. As mentioned, our results indicate that at least some of the last 33 residues of GN1 are critical for the interaction with TRIM7^B30.2^. However, the three-dimensional structure of full length GN1 (333 amino acids long) has not yet been solved by any experimental technique, and most of the available structures of the enzyme correspond to a 31 kDa truncated form lacking the last 71 amino acids. The only known crystal structure of the C-terminal domain is that of a peptide comprising the last 36 amino acids of *Caenorhabditis elegans* glycogenin in complex with glycogen synthase (PDB ID 4QLB (41)). The absence of a known structure for the remaining residues prevented any attempt to build a reliable full-length model of GN1. Consequently, based on the structure of *C. elegans,* we obtained a model including only the last 35 residues of GN1 (GN1-Cter) that was used to predict the structure of a complex with TRIM7^B30.2^ using HDOCK algorithm for protein–protein docking (42) (Fig. 8*A*).

Using the complex TRIM7^B30.2^/GN1-Cter built with HDOCK as a starting point, a 750 ns MD production run was performed. The stability of the complex during the simulation was measured by the RMSD of the backbone atoms. The modeled GN1-Cter structure consists of two α-helices joined by a loop (Fig. 8*A*). The α-helix composed of residues 299 to 311 (α-helix A) achieved convergence after 350 ns, while the other α-helix (α-helix B), formed by the last 12 residues, converged to a position that is close to that of the docking result after 100 ns, with an average RMSD value of approximately 3 Å (Fig. 8*B*). In this case, a peak in RMSD was observed in the interval from 200 ns to 350 ns, attributed to a transient conformational change of the last 2 residues of GN1-Cter, that then remained stable for the rest of the simulation. A comparison between the converged (snapshot at 700 ns) and the starting conformation (docking result) of GN1-Cter shows that α-helix B undergoes a slight change in its original position with respect to TRIM7^B30.2^, whereas α-helix A experiences a major conformational change before stabilization occurs (Fig. 8*A*). To identify the most important interactions between GN1-Cter and TRIM7^B30.2^, a contact map was calculated using the converged structure of the complex as a reference. Figure 8*C* shows the contact pattern (normalized and expressed as percentage of contact time between a residue-residue pair) mapped onto TRIM7^B30.2^ surface using a representative coloring scheme. TRIM7^B30.2^ residues displaying the highest contact time values are Cys501, Leu423, and Arg354, whereas in GN1-Cter, the interactions mainly involve Trp308, Asp319, Phe321, Ile324, Lys327, Leu328, and Tyr331 (Fig. 8*D*). GN1-Cter Phe321 locates inside the TRIM7^B30.2^ central pocket interacting with Cys501 and, to a lesser extent, with Ala410, Asn436 and Gln438. This result is more clearly seen in the analysis of the distances between the side chains of these TRIM7^B30.2^ residues with GN1-Cter Phe321 (Fig. S8*A*). In the crystal structure, TRIM7^B30.2^ Leu423 is highly exposed to the solvent, with a solvent accessible surface area (SASA) of approximately 270 Å^2^. In the complex with GN1-Cter, the SASA value of Leu423 decreases to about 50 Å^2^, and during MD simulations, this value finally converges to approximately 20 Å^2^ (Fig. S8*B*). The reduction of the SASA value suggests that Leu423 participates in the complex formation, and a detailed analysis of the structure reveals that it is surrounded by Ile324, Lys327, Leu328, and Tyr331 of GN1-Cter (Fig. 8*D*). All of these residues remain mostly at about 4 Å from Leu423 during the simulation, with the exception of Tyr331 (Fig. S8*C*). However, the most important interactions seem to involve Arg354 of TRIM7^B30.2^. This residue establishes a cation-π interaction with Trp308 of GN1-Cter from 200 ns and stable hydrogen bonding interactions with Asp319 after 100 ns of MD simulation (Fig. S8*D*). The analysis of the distance to Trp308 is consistent with the decrease in the SASA of Arg354 (Fig. S8*B*). The hydrogen bond interaction with Asp319 is the strongest interaction established with GN1-Cter, because it exhibits the highest occurrence frequency (95% of the simulation time) among all the observed contacts (Fig. S8*D*). Other important hydrogen bonding interactions involve Arg369 and Asp407 of TRIM7^B30.2^ and Glu326 and Tyr332 of GN1-Cter, respectively, with approximately 40% occurrence (not shown).

## Discussion

Members of the TRIM family of proteins are involved in a great variety of cellular processes. In particular, TRIM7 was initially identified through its interaction with GN1, the protein that initiates glycogen biosynthesis, hence its name GNIP. In addition to enhancing the activity of GN1, TRIM7 has E3 ubiquitin ligase activity (14), and through this function, it participates in several processes including glycogen accumulation (20), cancer development (14, 15, 16, 17), regulation of atherosclerosis (18), and of the toll-like receptor 4–mediated innate response (23). The E3 ubiquitin ligase function is also involved in the immune response against viral infections (20, 21, 22).

From a structural point of view, TRIM7 has the tripartite motif that characterizes TRIM proteins, constituted by a RING domain, a B-box domain, and a CC region, and at the C-terminal end, a B30.2 domain. This latter domain is responsible for the interaction with GN1 (13) and Src (16), and it is essential for the antiviral activity of the protein (21). According to the PROSITE database, this domain is present in more than 300 eukaryotic proteins and is likely to function as a protein-binding module (43). It is therefore conceivable that in addition to interacting with GN1 and Src, TRIM7^B30.2^ also participates in the interaction with other proteins and in this way mediates the different effects related to TRIM7. Among these proteins are probably RACO-1 (14), dual specificity phosphatase 6 (15), breast cancer metastasis suppressor 1 (17), the envelope protein of Zika virus (20), and mediator of IRF3 activation (22), all of them targets of TRIM7-mediated ubiquitination. Moreover, increased levels of TRIM7 correlate with the size of lung and liver tumors; interestingly, according to the Catalogue of Somatic Mutations in Cancer database of tumors from cancer patients (44), approximately 54% of the mutations associated with the TRIM7 gene are located in the B30.2 domain (Fig. S9), which includes 34% of the protein amino acids. Taken together, these data reflect the potential relevance of having structural information at the atomic level of this domain.

In the present article, we report on the X-ray crystal structure of TRIM7^B30.2^, which constitutes the first structural study of any portion of this protein. The structure of TRIM7^B30.2^ exhibits a high homology with the B30.2 domains of other proteins, the most homologous being that of BTN3A1, which curiously does not belong to the TRIM family. A primary and tertiary structure conservation analysis revealed that the least conserved residues of TRIM7^B30.2^ are mostly located on the concave side of the β-sandwich including loops 3, 6, 8, and 13. Because B30.2 domains are structurally very similar, it can be assumed that the difference in their specificity lies, at least in part, in these less conserved amino acids. Similar hypotheses have also been proposed by others (29, 38, 40, 45). In agreement with this analysis, in the B30.2 domain structure of other proteins that were crystallized with a binding partner (mostly short peptides) there appear to be two zones involved in the interaction, one in the region equivalent to loops 3 and 13 of TRIM7^B30.2^ (observed in the structures of the complexes GUSTAVUS/VASA peptide (46), suppressor of cytokine signaling (SOCS)1/VASA peptide, SOCS1/Par4 and SOCS2/VASA peptide (47)), and another on the concave surface of the β-sandwich (found in the structures of TRIM21^B30.2^/IgG (38), TRIM65^B30.2^/MDA5 (31), BTN3A1/phosphoantigen (32), and Ash2L/RbBP5 (48)).

In the case of TRIM7^B30.2^, one of the known binding partners is GN1, and therefore, this protein was used in the binding assays with mutants of TRIM7^B30.2^ to define potentially important residues for the interaction. This study revealed that mutations Leu423Ala and Ser499A interfered with the interaction between the proteins, whereas the substitution of Arg354 produced an increase in binding affinity. The replacement of the nearby Cys501 by a serine, which is modified by β-mercaptoethanol in the crystal structure, had a similar effect to that observed in Leu423Ala and Ser499Ala on the binding to GN1, indicating that it is also a critical residue for the interaction. Together, these data suggest that the interaction with the enzyme involves the concave surface of TRIM7^B30.2^ and that the pocket in which malonate is bound (Fig. 2*B*) could play an important role in such interaction. This pocket would be a good candidate as a binding site for inhibitors of the interaction with GN1 and perhaps with other substrates as well. Moreover, the presence of Cys501 could even be exploited for the binding of cysteine-targeted irreversible inhibitors.

Our findings indicate that the final 33 amino acids of GN1 are important for the binding to TRIM7^B30.2^. This portion of the protein is also involved in the interaction with glycogen synthase (49) from which it can be assumed that whereas the N-terminal domain, common to the glycosyltransferase family 8, is responsible for the catalytic activity of the protein, the function of the C-terminal region might be to interact with other proteins. The only known three-dimensional structure of this region is that of the C-terminal end of *C. elegans* glycogenin in complex with glycogen synthase (41), which spans residues 394 to 428 of the protein (complete sequence length: 429 amino acids). These are equivalent to the last human GN1 residues with which they share an identity of 48.5% and a similarity of 72.7%. This terminal region is bound to the catalytically active globular domain through a poorly conserved linker sequence that could confer some structural independence. With this in mind, a homology model of human GN1 C-terminal region was generated using the structure of the same region of *C. elegans* glycogenin as a template. The complex built by docking this model into the crystal structure of TRIM7^B30.2^ was analyzed by MD simulations. According to this study, the complex is stable on the simulation timescale. A detailed analysis of the results indicates that residues Cys501, Leu423, and Arg354 of TRIM7^B30.2^ (three of the four residues that, when mutated, alter the binding to GN1) participate in the interaction with GN1-Cter. As expected, the positive pocket mentioned above is involved in the binding, and in this region, the most important interaction is the thiol-π-type interaction between Cys501 and Phe321 of GN1-Cter. Leu423 forms hydrophobic interactions with several amino acids, whereas Arg354 interacts strongly with Asp319 and Trp308 of GN1-Cter. If this were the case, the enhanced binding to GN1 when Arg354 is replaced by alanine could be explained by the appearance of new interactions in the absence of this bulky residue and to the formation of hydrophobic interactions involving alanine, for example, with Trp308. On the other hand, the side chain of Ser499 forms a strong hydrogen bond with the main chain amide nitrogen of Thr384, which is also located within the central pocket of TRIM7^B30.2^. Therefore, the replacement of Ser499 by an alanine could alter the structure of the pocket and, consequently, hinder the hydrophobic interactions between GN1-Cter and TRIM7^B30.2^.

As mentioned before, TRIM7 is also able to interact with RACO-1 and more specifically with its C-terminal end. However, the region of TRIM7 involved in this interaction is unknown. Interestingly, the sequence alignment of the C-terminal ends of GN1 and RACO-1 revealed that they exhibit 18.4% identity and 68.5% similarity (Fig. S10). This, together with the known protein binding properties of the B30.2 domains, reinforce the hypothesis that TRIM7 could also interact with RACO-1 through this domain. Moreover, we can also speculate that, like RACO-1, GN1 might be a substrate of TRIM7-mediated ubiquitination. However, because approximately 90% of GN1 is covalently bound to glycogen (50) and therefore not accessible to TRIM7, ubiquitination might have some relevance in the case of pathological mutants of the enzyme, which because of their inactivity are free of polysaccharide (51, 52, 53, 54).

During protein ubiquitination, E3 ubiquitin ligases have the crucial role of determining the substrate specificity of the reaction. For this reason, together with the fact that they are implicated in a wide variety of human diseases, they have become attractive therapeutic targets. Whereas the E3 ubiquitin ligase function of TRIM7 and other TRIM proteins depends on the presence of the RING domain, which interacts with the E2 ubiquitin-conjugating enzyme, the specificity of the ubiquitination reaction is determined by the C-terminal domain (B30.2 domain in the case of TRIM7). Modulators of these enzymes targeting the B30.2 domain are therefore expected to have more selectivity and less side-effects. In this context, because TRIM7 is involved in carcinogenesis and viral pathogenesis, the three-dimensional structure of TRIM7^B30.2^ and the information related to its interaction with target proteins could be relevant for the development of potential therapeutic agents.

## Experimental procedures

### Materials

*Escherichia coli* strain Rosetta(DE3) and vector pET-15b were purchased from Novagen. pTYB11 vector and chitin agarose beads were obtained from New England Biolabs. The codon optimized cDNA encoding human TRIM7 was synthesized by GenScript (GenScript Inc). Protease Inhibitor Mix, HisTrap HP, and Superdex 200 10/300 GL columns were from GE Healthcare. Primary anti-His_6_ tag antibody was from Invitrogen, and dye-labeled secondary antibody was from LI-COR. Except where indicated, all other reagents were from Sigma-Aldrich.

### Protein mutagenesis, expression, and purification

The cDNA of human TRIM7^B30.2^ (amino acids 338–511) was subcloned into the pET-15b vector downstream of the His_6_-tag encoding sequence of the plasmid. The resulting construct was used as template to generate the mutants TRIM7^B30.2^ Arg354Ala, TRIM7^B30.2^ Glu368Ala, TRIM7^B30.2^ Thr384Ala, TRIM7^B30.2^ Leu423Ala, TRIM7^B30.2^ Asn438Ala, TRIM7^B30.2^ Ser499Ala, and TRIM7^B30.2^ Cys501Ser by site-directed mutagenesis utilizing the QuickChange site-directed mutagenesis kit (Agilent Technologies). The cDNA of human GN1 was also subcloned in pET-15b to obtain the N-terminal His_6_-tagged protein. The cDNA of full-length human TRIM7 was subcloned into the pGEX-4T-1 downstream of the GST encoding sequence of the plasmid.

His_6_-human GN1, His_6_-human TRIM7^B30.2^, and its mutants were expressed in the *E. coli* Rosetta(DE3) strain. Protein expression was induced at OD_600_ 0.6 to 1.0 overnight with 0.15 mM isopropyl β-D-thiogalactopyranoside at 18 °C. The proteins were purified from clarified cell lysates by affinity chromatography on HisTrap HP columns (1 ml), and TRIM7^B30.2^ was further subjected to gel-filtration chromatography on a Superdex 200 column in 20 mM Tris-HCl buffer pH 7.5, 150 mM NaCl, and 10 mM β-mercaptoethanol. Fractions containing the protein of interest were pooled and desalted with 20 mM Tris-HCl buffer pH 7.5, 10 mM β-mercaptoethanol. Protein concentration was determined based on calculated extinction coefficients at 280 nm of 56,380 M^−1^ cm^−1^ and 33,460 M^−1^ cm^−1^ for GN1 and TRIM7^B30.2^, respectively.

### Crystallization, data collection, and structure determination

Purified TRIM7^B30.2^ was concentrated to 5 mg/ml in 20 mM Tris-HCl buffer pH 7.5, 10 mM β-mercaptoethanol, and 1 mM tris (2-carboxyethyl) phosphine for the crystallization setup. Initial crystallization trials were performed by the hanging-drop vapor diffusion method at 10 °C using commercially available crystallization screens from Qiagen (Classics, Classics II, PACT and Protein Complex Suites). Small crystals were observed within a week in 1.4 M sodium malonate pH 6.0. During optimization, larger crystals were obtained in the same condition but using the sitting-drop vapor diffusion method and by mixing equal volumes of the protein solution at 10 mg/ml and 1.4 M sodium malonate pH 6.0 supplemented with glycerol 3% using the hanging-drop vapor diffusion method.

Crystals were cryoprotected by soaking in a solution containing 85% mother liquor and 15% glycerol before being flash-frozen. Data were collected at the Macromolecular Crystallography beamline MX2 (wavelength 1.4586 Å) of the Laboratório Nacional de Luz Síncrotron (LNLS; Campinas) at 100 K.

Diffraction data were integrated and scaled using iMOSFLM (55) and AIMLESS (56) from the CCP4 suite of programs for crystallographic computing (57). Molecular replacement using the B30.2 domain of Butyrophilin 3A1 (PDB ID 4N7I, chain A) as a search model was done using PHASER (58). The resulting models were iteratively improved by cycles of manual rebuilding and refinement performed with COOT (59) and REFMAC5 (60), respectively. Solvent molecules and ligands were added to the models in the final stages of refinement based on examination of the difference density maps. Data collection and refinement statistics are reported in Table 1. Structure comparisons were done with Dali server (25); crystal packing and quaternary structure were analyzed with PISA (33) and direct contacts with Ligplot (61). Amino acid sequences were aligned by Clustal Omega (36) and ConSurf was used for the analysis of sequence conservation (37). DSSP was used to assign the secondary structure elements (62). Figures were generated using PyMOL (Schrödinger LLC; http://www.pymol.org), and electrostatic surface calculations were performed using the APBS plugin for PyMOL (63).

### Circular dichroism measurements

CD spectra of TRIM7^B30.2^ variants (2.5 μM) were collected using a JASCO J810 spectropolarimeter (Jasco) in 0.2 cm path-length quartz cuvettes. The spectra represent the average of five scans recorded in the far-ultraviolet region (190–260 nm) with a bandwidth of 2.0 nm, a step size of 0.2 nm, and a response time of 8 s. The measurements were corrected by subtracting signals from the buffer control.

### Pull-down assays

To obtain N-terminal CBD–intein–tagged human GN1, its cDNA was subcloned in the vector pTYB11, while the truncated variants (GN1ΔC63 and GN1ΔC33) were obtained by introducing a stop codon in the corresponding position by site-directed mutagenesis. Intein used as control was prepared from the empty plasmid. Intein and the different intein–GN1 variants were expressed in the *E. coli* Rosetta(DE3) strain and immobilized on chitin beads from the bacterial clarified lysate followed by three washes with phosphate-buffered saline (PBS buffer). Full-length TRIM7, TRIM7^B30.2^, and all the mutants (Arg354Ala, Glu368Ala, Thr384Ala, Leu23Ala, Asn438Ala, Ser499Ala, and Cys501Ser) were obtained in the same *E. coli* strain. Even though their expression levels were very similar, to ensure the use of equivalent amounts of all the TRIM7^B30.2^ variants, the clarified extracts were analyzed by immunoblot with an anti-His_6_ tag antibody, and the relative intensity of the bands was quantified using ImageJ software. Twenty microliter of the chitin beads carrying similar molar amounts of intein or intein–GN1 variants were incubated at 4 °C for 2 h with 50 μl of the extracts (equivalent to approximately 300 μl of bacterial culture) prepared in PBS buffer supplemented with a protease inhibitor mix. The beads were subsequently washed three times with PBS buffer, and the proteins were eluted by boiling at 100 °C for 5 min with SDS-PAGE sample buffer. Eluted TRIM7^B30.2^ variants were detected by immunoblot analysis on a polyvinylidene fluoride membrane with a primary anti-His_6_ tag antibody and IRDye 800 goat anti-mouse IgG secondary antibody, using the Odyssey Infrared Imaging System (LI-COR). In the case of the pull-down assay using TRIM7, the chitin beads carrying similar molar amounts of intein or intein–GN1 variants were incubated at 4 °C for 2 h with 50 μl of the extract prepared in 20 mM Tris-HCl buffer pH 7.5, 150 mM NaCl, 0.5% Triton X-100, 100 μM PMSF, 5 mM β-mercaptoethanol, and 10% glycerol (14) and washed with the same buffer. TRIM7 was detected with a primary anti-TRIM7 antibody (Santa Cruz Biotechnology) and the secondary IRDye 680 donkey anti-rabbit IgG antibody. After immunodetection, the membranes were washed with Tris-buffered saline with Tween solution (20 mM Tris-HCl, pH 7.5, 150 mM NaCl, and 0.2% Tween-20) for several hours, stained with 0.1% Coomassie Brilliant blue for 1 min, destained in 50% ethanol/10% acetic acid solution, and finally washed with water and air-dried.

### Protein–protein docking

Human GN1 C-terminal peptide structure was modeled with MODELLER (64), using as a template the structure reported for *C. elegans* glycogenin C-terminal peptide (residues 394–428) in complex with glycogen synthase (PDB ID 4QLB, chain E (41)). The three-dimensional structure of the complex between human TRIM7^B30.2^ (PDB ID 6UMA, chain A) and the modeled C-terminal peptide of human GN1 was predicted using HDOCK docking algorithm (42). Docking calculations were performed with default parameters using the web server version of the algorithm (http://hdock.phys.hust.edu.cn/).

### Molecular dynamic simulations

The complex between TRIM7^B30.2^ and GN1 C-terminal peptide was placed into a truncated octahedral box of TIP3P water molecules, with a distance of 15 Å between the border of the box and the closest atom of the solute. The total system is composed of 207 protein residues, with 11,707 water molecules, giving a total of 38,403 atoms. The AMBER14SB force field was chosen to assign the parameters of all protein residues. All the systems were optimized with an energy minimization step consisting of 10,000 cycles using the steepest descent algorithm and 10,000 cycles with conjugate gradient minimization. The temperature was increased from 0 to 10 K in a 10 ps constant volume MD with a 0.1 fs time step, and a harmonic restraint potential of 10 kcal mol^−1^ Å^−2^ applied over all protein residues in the complex. Thereafter, the temperature was increased from 10 to 300 K in a 50 ps constant volume MD with a 0.5 fs time step, applying a force constant of 5 kcal mol^−1^ Å^−2^ to the protein backbone atoms. After the samples had been heated, the density was equilibrated with a 100 ps MD simulation at constant temperature and pressure with a time step of 1 ps and applying a force constant of 1 kcal mol^−1^ Å^−2^ to the protein backbone atoms. To control the temperature, a Langevin thermostat was used, whereas a Berendsen barostat was chosen to adjust the pressure to 1 bar (both regulated every 1 ps). For production MD, 750 ns simulations in the NTP ensemble were conducted, with a time step of 2 fs. All the simulations were performed under periodic boundary conditions (65) using the SHAKE algorithm (66) to keep hydrogen atoms at equilibrium bond lengths. Long-range electrostatic interactions were handled with Ewald sums, setting a cutoff distance of 10 Å. The contact map between residues from TRIM7^B30.2^ and GN1 C-terminal peptide for the converged structure of the complex was done with *nativecontacts* utility implemented in Amber18, using the structure of the system at 450 ns MD as a reference. For the analysis of the trajectories (RMSD, distance analysis, contact map), cpptraj (67) from AMBER18 was used. All molecular visualization and drawings were performed with the Visual Molecular Dynamics program (68).

## Data availability

The atomic coordinates and structure factors have been deposited in the Protein Data Bank under accession numbers 6UMA (1.6 Å resolution structure) and 6UMB (1.8 Å resolution structure). All remaining data are available in the main text and the supporting information or from the corresponding author upon request.

## Supporting information

This article contains supporting information (32, 36, 44, 62, 69).

## Conflict of interest

The authors declare that they have no conflicts of interest with the contents of this article.
