# Supplementary material for: Crystal structure and mutational analysis of the human TRIM7 B30.2 domain provide insights into the molecular basis of its binding to glycogenin-1
Source: J Biol Chem. 2021 May 11;296:100772. doi: 10.1016/j.jbc.2021.100772 (PMC8203840; doi:10.1016/j.jbc.2021.100772)
Supplement: Figures S1 to S10 [file mmc1.pdf]

Supporting Information

Crystal structure and mutational analysis of the human TRIM7 B30.2 domain provide insights into the molecular basis of its binding to glycogenin-1

Christian J. Muñoz Sosa[1], Federico M. Issoglio[2,3] and María E. Carrizo[1]*

[1] Centro de Investigaciones en Química Biológica de Córdoba (CIQUIBIC) - CONICET and Departamento de Química Biológica Ranwel Caputto, Facultad de Ciencias Químicas, Universidad Nacional de Córdoba, X5000HUA Córdoba, Argentina

[2] Instituto de Tecnologia Química e Biológica António Xavier, Universidade Nova de Lisboa (ITQB NOVA), 2780-157 Oeiras, Portugal

[3] Instituto de Química Biológica de la Facultad de Ciencias Exactas y Naturales (IQUIBICEN) - CONICET and Departamento de Química Biológica, Facultad de Ciencias Exactas y Naturales, Universidad de Buenos Aires, C1428EGA, Buenos Aires, Argentina

* For correspondence: María E. Carrizo; ecarrizo@fcq.unc.edu.ar, mariae.carrizo@unc.edu.ar.

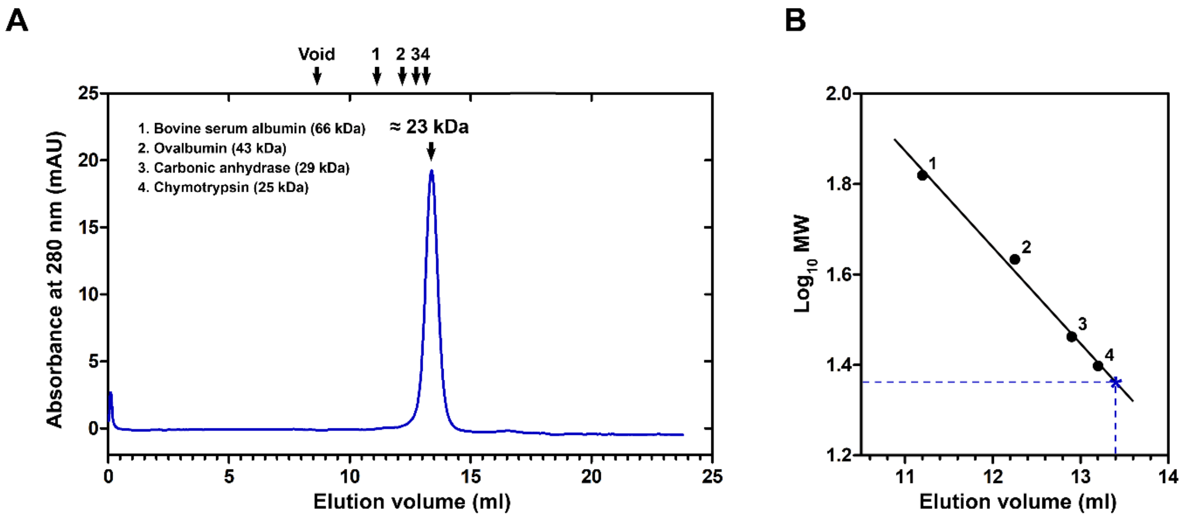

Figure S1. Size exclusion chromatography profile of TRIM7$^{B30.2}$. (A) TRIM7$^{B30.2}$ was analyzed on a Superdex 75 10/300 GL column. Arrows indicate the elution volumes of marker proteins. The molecular weight of TRIM7$^{B30.2}$ was estimated to be approximately 23 kDa, consistent with a monomeric state in solution. (B) Relation between elution volume and molecular mass of the marker proteins in gel filtration analysis. mAU, milliabsorbance units.

```
                            ▼▼▼▼▼  ▼▼▼                ▼
BTN3A1    LFKPADVILDPKTANPILLVSEDQRSVQRAKEPQDLPDNPERFNW   350
            : .:: ***.**** *::* * :.*: .:. ****::* **:
TRIM7     KEEKVELTLDPDTANPRLILSLDLKGVRLGERAQDLPNHPCRFDT   382

           ▼                                      ▼
BTN3A1    HYCVLGCESFISGRHYWEVEVGDRKEWHIGVCSKNVQRKGWVKMT   395
          :  **.. .* ****:******.:. * :**. :.*:*** . :*
TRIM7     NTRVLASCGFSSGRHHWEVEVGSKDGWAFGVARESVRRKGLTPFT   427

BTN3A1    PENGFWTMGLTDGNKYRTLTEPRTNLKLPKPPKKVGVFLDYETGD   440
          **:*.*:: *..*: : . :  *: *      .:* * ** *.*
TRIM7     PEEGVWALQLNGGQYWAVTSPERSPL-SCGHLSRVRVALDLEVGA   471

                                              ▼
BTN3A1    ISFYNAVDGSHIHTFLDVSFSEALYPVFRILTLEPTALTICPA     483
          :*** . *  *::**  *.*.* ::*:* : :   * * * *
TRIM7     VSFYAVEDMRHLYTF-RVNFQERVFPLFSVCSTG-TYLRIWP-     511
```

Identity: 35.7% (5/14 residues); similarity: 42.9% (6/14 residues)

Identity: 0% (0/7 residues); similarity: 28.6% (2/7 residues)

Figure S2. Sequence alignment of TRIM7 and BTN3A1 B30.2 domains. BTN3A1$^{B30.2}$ amino acids involved in the interaction between protomers in Dimer I (32) and their homologs in TRIM7$^{B30.2}$ are boxed. Residues colored in yellow and blue are from different protomers of the dimer. Triangles indicate residues that maintain stable contacts within the dimer according to molecular dynamics simulations. Conserved amino acids are indicated by an asterisk (*****), strong amino acid group similarity by a colon (**:**) and weaker group similarity by a single dot (**.**). The color code indicates the different types of amino acids according to Clustal Omega (36).

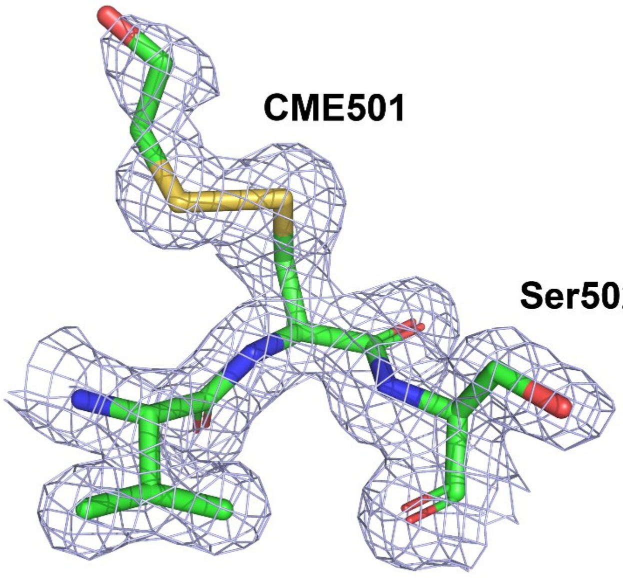

Figure S3. TRIM7$^{B30.2}$ Cys501 modification by β-mercaptoethanol. 2F_o-F_c electron density map at 1.6 Å of the vicinity of Cys501 contoured at 1σ. The cysteine is modified as S,S-(2-hydroxyethyl)thiocysteine (CME).

Consurf Color-Coded Multiple Sequence Alignment

```
001 TRIM14|Q14142|249-442     --------TLLKTSPSPERSLLLKYARTPTLDPDTMHARLRLSADRLTVR
002 TRIM47|Q96LD4|410-631     -------STNLLE-SEAPRDYFLKFAYIVDLDSDTADKFLQLFGTKG--V
003 TRIM49|P0CI25|269-452     ELS-AGPITG-------LRDRLNQFRVHITLHHEEANNDIFLYEILRSMC
004 TRIM16|O95361|355-553     -------YWTSKP-EPSTREQFLQYAYDITFDPDTAHKYLRLQEENRKVT
005 TRIM17|Q9Y577|277-475     -PRTVCRVPGQIEVL-------RGFLEDVVPDATSAYPYLLLYESRQRRY
006 TRIM27|P14373|298-492     MQSDMEKIQELRE-A-------QLYSVDVTLDPDTAYPSLILSDNLRQVR
007 TRIM72|Q6ZMU5|271-475     ----DFKFQVWR----KMFRALMPALEELTFDPSSAHPSLVVSSSGRRVE
008 TRIM15|Q9C019|276-465     --------EMMRMFSENLAHHLEIDSGVITLDPQTASRSLVLSEDRKSVR
009 TRIM22|Q8IYM9|283-498     -LSGM-----LQVL--KELTDVQYYWVDVMLNPGSATSNVAISVDQRQVK
010 TRIM10|Q9UDY6|292-481     --------REMKMFLEKLCFELDYEPAHISLDPQTSHPKLLLSEDHQRAQ
011 TRIM51|Q9BSJ1|269-452     ELS-AGPITG-------LLDSLSGFRVDFTLQPERANSHIFLCGDLRSMN
012 TRIM41|Q8WV44|413-630     -LT-DAIVRK----MS--RMFCQAARVDLTLDPDTAHPALMLSPDRRGVR
013 TRIM11|Q96F44|268-461     ELRTVCRVPGLVETL-------RRFRGDVTLDPDTANPELILSEDRRSVQ
014 TRIM7|Q9C029|324-511      ------MLKKFKEDLR--GELEKEEKVELTLDPDTANPRLILSLDLKGVR
015 RNF39|Q9H2S5|210-420      --------DDLPEDYPVVKKMLHRLTADLTLDPGTAHRRLLISADRRSVQ
016 TRIM68|Q6AZZ1|285-481     -LKTDCRVLGLREIL-------KTYAADVRLDPDTAYSRLIVSEDRKRVH
017 TRIM39|Q9HCM9|319-514     -SNFPRQYFALRKIL-------KQLIADVTLDPETAHPNLVLSEDRKSVK
018 TRIM60|Q495X7|277-470     -FSLPPQYSGLDRII-------KPFQVDVILDLNTAHPQLLVSEDRKAVR
019 TRIM43|Q96BQ3|269-446     ELT-AGPITG-------LVYRLNRFRVEISFHFEVTNHNIRLFEDVRSWM
020 PYRIN|O15553|580-775      SEMEMFNVPELIG-A-------QAHAVNVILDAETAYPNLIFSDDLKSVR
021 TRIM69|Q86WT6|305-500     ----PIQYMVWREMQ----DTLCPGLSPLTLDPKTAHPNLVLSKSQTSVW
022 ERMAP|Q96PL5|220-418      KLKRAAANSGWRR-A-------RLHFVAVTLDPDTAHPKLILSEDQRCVR
023 TRIM75P|A6NK02|276-468    -CSFPPQYSALQRII-------KKFKVEIILDPETAHPNLIVSEDKKRVR
024 TRIM35|Q9UPQ4|284-487     --LGSLQYRVWKKMLA------SVESVPFSFDPNTAAGWLSVSDDLTSVT
025 TRIM77|I1YAP6|269-450     QLS-AWTITG-------VSERLNFFRVYITLDRKICSNHKLLFEDLRHLQ
026 TRIM9|Q9C026|533-702      -----------------------SEVAWFAFDPGSAHSDIILSNDNLTVT
027 TRIM65|Q6PJ69|313-506     --------APVPSTVCPLRRKLWQNYRNLTFDPVSANRHFYLSRQDQQVK
028 TRIM62|Q9BVG3|277-475     ----PLQYTIWKSLF----QDIHPVPAALTLDPGTAHQRLILSDDCTIVA
029 TRIM38|O00635|274-465     -----CNVSKLYFDV---KKMLRSHQVSVTLDPDTAHHELILSEDRRQVT
030 BTN1A1|Q13410|285-479     ------SKERLLEELK--WKKATLHAVDVTLDPDTAHPHLFLYEDSKSVR
031 BTN3A1|O00481|322-513     ------RGERHSAYNE--WKKALFKPADVILDPKTANPILLVSEDQRSVQ
032 TRIM25|Q14258|439-630     KV-----LETFLA-KSRP--ELLEYYIKVILDYNTAHNKVALSECYTV-A
033 TRIM26|Q12899|295-539     --------RGLREFQGKLLRDLEYKTVSVTLDPQSASGYLQLSEDWKCVT
034 TRIM58|Q8NG06|273-463     -LKTACCIPGRRELL-------RKFQVDVKLDPATAHPSLLLTADLRSVQ
035 TRIM6|Q9C030|282-488      DLKRM-----LRVC--RELTDVQSYWVDVTLNPHTANLNLVLAKNRRQVR
036 TRIM64|A6NGJ6|269-449     -LT-SWCITG-------VLDMLNNFRVDSALSTEMIPCYISLSEDVRYVI
037 TRIM21|P19474|268-465     ELRSVCHVPGLKKML-------RTCAVHITLDPDTANPWLILSEDRRQVR
038 BTN2A1|Q7KYR7|310-506     ------VKEKLQEELR--WRRTFLHAVDVVLDPDTAHPDLFLSEDRRSVR
039 TRIM67|Q6ZTA4|589-780     NSSGVGPYSKT-------VVLQTSDVAWFTFDPNSGHRDIILSNDNQTAT
040 TRIM34|Q9BYJ4|283-488     -LSRM-----LQMF--RELTAVRCYWVDVTLNSVNLNLNLVLSEDQRQVI
041 TRIM50|Q86XT4|276-475     ----DIKLTVWK----RLFRKVLPAPEPLKLDPATAHPLLELSKGNTVVQ
042 TRIM4|Q9C037|288-500      KVKTVCQIPLMKEML-------KRFQVAVNLAEDTAHPKLVFSQEGRYVK
```

```
001 TRIM14|Q14142|249-442     CGLL---G---------------SLGPVPVLRFDALWQVLARDCFATGRH
002 TRIM47|Q96LD4|410-631     KRVL---CP--------------INYPLSPTRFTHCEQVLGEGALDRGTY
003 TRIM49|P0CI25|269-452     IGCD---H---------------QDVPYFTATPR-SFLAWGVQTFTSGKY
004 TRIM16|O95361|355-553     NTTP---WE--------------HPYPDLPSRFLHWRQVLSQQSLYLHRY
005 TRIM17|Q9Y577|277-475     LGSS--PE---------------GSGFCSKDRFVAYPCAVGQTAFSSGRH
006 TRIM27|P14373|298-492     YSY---LQ---------------QDLPDNPERFNLFPCVLGSPCFIAGRH
007 TRIM72|Q6ZMU5|271-475     CSEQ--KA---------------PPAGEDPRQFDKAVAVVAHQQLSEGEH
008 TRIM15|Q9C019|276-465     YTRQ---K---------------KSLPDSPLRFDGLPAVLGFPGFSSGRH
009 TRIM22|Q8IYM9|283-498     TVRT---C---------------TFKNSNPCDFS-AFGVFGCQYFSSGKY
010 TRIM10|Q9UDY6|292-481     FSYK---W---------------QNSPDNPQRFDRATCVLAHTGITGGRH
011 TRIM51|Q9BSJ1|269-452     VGCD---P---------------QDDPDITGKSE-CFLVWGAQAFTSGKY
012 TRIM41|Q8WV44|413-630     LAER---R---------------QEVADHPKRFSADCCVLGAQGFRSGRH
013 TRIM11|Q96F44|268-461     RGDL---R---------------QALPDSPERFDPGPCVLGQERFTSGRH
014 TRIM7|Q9C029|324-511      LGER---A---------------QDLPNHPCRFDTNTRVLASCGFSSGRH
015 RNF39|Q9H2S5|210-420      LAPP--GT---------------PAPPDGPKRFDQLPAVLGAQGFGAGRH
016 TRIM68|Q6AZZ1|285-481     YGDT---N---------------QKLPDNPERFYRYNIVLGSQCISSGRH
017 TRIM39|Q9HCM9|319-514     FVET--RL---------------RDLPDTPRRFTFYPCVLATEGFTSGRH
018 TRIM60|Q495X7|277-470     YERK---K---------------RNICYDPRRFYVCPAVLGSQRFSSGRH
019 TRIM43|Q96BQ3|269-446     FRR----------------------GPLNSDRSD-YFAAWGARVFSFGKH
020 PYRIN|O15553|580-775      LGN---KW---------------ERLPDGPQRFDSCIIVLGSPSFLSGRR
021 TRIM69|Q86WT6|305-500     HGDI---K---------------KIMPDDPERFDSSVAVLGSRGFTSGKW
022 ERMAP|Q96PL5|220-418      LGD---RR---------------QPVPDNPQRFDFVVSILGSEYFTTGCH
023 TRIM75P|A6NK02|276-468    FTKR---K---------------QKVPGFPKRFTVKPVVLGFPYFHSGRH
024 TRIM35|Q9UPQ4|284-487     NHGY-------------------RVQVENPERFSSAPCLLGSRVFSQGSH
```

```
025 TRIM77|I1YAP6|269-450   CSLD---D---------------TDMSCNPTSTQ-YTSSWGAQILSSGKH
026 TRIM9|Q9C026|533-702    CSSY---D---------------------------DRVVLGKTGFSKGIH
027 TRIM65|Q6PJ69|313-506   HCRQ-------------------SRGPGGPGSFE-LWQVQCAQSFQAGHH
028 TRIM62|Q9BVG3|277-475   YGNL--HP---------------QPLQDSPKRFDVEVSVLGSEAFSSGVH
029 TRIM38|O00635|274-465   RGYT---Q---------------ENQDTSSRRFTAFPCVLGCEGFTSGRR
030 BTN1A1|Q13410|285-479   LEDS---R---------------QKLPEKTERFDSWPCVLGRETFTSGRH
031 BTN3A1|O00481|322-513   RAKE---P---------------QDLPDNPERFNWHYCVLGCESFISGRH
032 TRIM25|Q14258|439-630   SVAE---MP--------------QNYRPHPQRFTYCSQVLGLHCYKKGIH
033 TRIM26|Q12899|295-539   YTSL---Y---------------KSAYLHPQQFDCEPGVLGSKGFTWGKV
034 TRIM58|Q8NG06|273-463   DGE---PW---------------RDVPNNPERFDTWPCILGLQSFSSGRH
035 TRIM6|Q9C030|282-488    FVGA---K---------------VSGPSCLEKHY-DCSVLGSQHFSSGKH
036 TRIM64|A6NGJ6|269-449   FGDD---H---------------LSAPTDPQGVD-SFAVWGAQAFTSGKH
037 TRIM21|P19474|268-465   LGDT---Q---------------QSIPGNEERFDSYPMVLGAQHFHSGKH
038 BTN2A1|Q7KYR7|310-506   RCPFRHLG---------------ESVPDNPERFDSQPCVLGRESFASGKH
039 TRIM67|Q6ZTA4|589-780   CSSY---D---------------------------DRVVLGTAAFSKGVH
040 TRIM34|Q9BYJ4|283-488   SVPI---W---------------PF------QCY-NYGVLGSQYFSSGKH
041 TRIM50|Q86XT4|276-475   CGLL--A----------------QRRASQPERFDYSTCVLASRGFSCGRH
042 TRIM4|Q9C037|288-500    NTASASSWPVFSSAWNYFAGWRNPQKTAFVERFQHLPCVLGKNVFTSGKH
```

```
001 TRIM14|Q14142|249-442   YWEVDVQE------------------------------------------
002 TRIM47|Q96LD4|410-631   YWEVEIIEG-----------------------------------------
003 TRIM49|P0CI25|269-452   YWEVHV--G-----------------------------------------
004 TRIM16|O95361|355-553   YFEVEIFGA-----------------------------------------
005 TRIM17|Q9Y577|277-475   YWEVGMNIT-----------------------------------------
006 TRIM27|P14373|298-492   YWEVEV--G-----------------------------------------
007 TRIM72|Q6ZMU5|271-475   YWEVDV--G-----------------------------------------
008 TRIM15|Q9C019|276-465   RWQVDLQLG-----------------------------------------
009 TRIM22|Q8IYM9|283-498   YWEVDV--S-----------------------------------------
010 TRIM10|Q9UDY6|292-481   TWVVSIDLA-----------------------------------------
011 TRIM51|Q9BSJ1|269-452   YWEVHM--G-----------------------------------------
012 TRIM41|Q8WV44|413-630   YWEVEV--G-----------------------------------------
013 TRIM11|Q96F44|268-461   YWEVEV--G-----------------------------------------
014 TRIM7|Q9C029|324-511    HWEVEV--G-----------------------------------------
015 RNF39|Q9H2S5|210-420    CWEVETADAASCRD------------------------------------
016 TRIM68|Q6AZZ1|285-481   YWEVEV--G-----------------------------------------
017 TRIM39|Q9HCM9|319-514   YWEVEV--G-----------------------------------------
018 TRIM60|Q495X7|277-470   YWEVEV--G-----------------------------------------
019 TRIM43|Q96BQ3|269-446   YWELDV--D-----------------------------------------
020 PYRIN|O15553|580-775    YWEVEV--G-----------------------------------------
021 TRIM69|Q86WT6|305-500   YWEVEV--A-----------------------------------------
022 ERMAP|Q96PL5|220-418    YWEVYV--G-----------------------------------------
023 TRIM75P|A6NK02|276-468  FWEIEV--G-----------------------------------------
024 TRIM35|Q9UPQ4|284-487   AWEVAL--G-----------------------------------------
025 TRIM77|I1YAP6|269-450   YWEVDV--K-----------------------------------------
026 TRIM9|Q9C026|533-702    YWELTVDRY-----------------------------------------
027 TRIM65|Q6PJ69|313-506   YWEVRASD------------------------------------------
028 TRIM62|Q9BVG3|277-475   YWEVVV--A-----------------------------------------
029 TRIM38|O00635|274-465   YFEVDV--G-----------------------------------------
030 BTN1A1|Q13410|285-479   YWEVEV--G-----------------------------------------
031 BTN3A1|O00481|322-513   YWEVEV--G-----------------------------------------
032 TRIM25|Q14258|439-630   YWEVELQKN-----------------------------------------
033 TRIM26|Q12899|295-539   YWEVEVEREGWSEDEEEGDEEEEGEEEEEEEEAGYGDGYDDWETDEDEES
034 TRIM58|Q8NG06|273-463   YWEVLV--G-----------------------------------------
035 TRIM6|Q9C030|282-488    YWEVDV--A-----------------------------------------
036 TRIM64|A6NGJ6|269-449   YWEVDV--T-----------------------------------------
037 TRIM21|P19474|268-465   YWEVDV--T-----------------------------------------
038 BTN2A1|Q7KYR7|310-506   YWEVEV--E-----------------------------------------
039 TRIM67|Q6ZTA4|589-780   YWELHVDRY-----------------------------------------
040 TRIM34|Q9BYJ4|283-488   YWEVDV--S-----------------------------------------
041 TRIM50|Q86XT4|276-475   YWEVVV--G-----------------------------------------
042 TRIM4|Q9C037|288-500    YWEVES--R-----------------------------------------
```

```
001 TRIM14|Q14142|249-442   --------------AGAGWWVGAAYASLRRRGASAA--------------
002 TRIM47|Q96LD4|410-631   -----------------WVSMGVMAEDFSPQEPYDR--------------
003 TRIM49|P0CI25|269-452   --------------DSWNWAFGVCNMYRKEKNQNEK--------------
004 TRIM16|O95361|355-553   -----------------GTYVGLTCKGIDRKGEERN--------------
005 TRIM17|Q9Y577|277-475   --------------GDALWALGVCRDNVSRKDRVPK--------------
006 TRIM27|P14373|298-492   --------------DKAKWTIGVCEDSVCRKGGVTS--------------
```

```
007 TRIM72|Q6ZMU5|271-475     --------------DKPRWALGVIAAEAPRRGRLHA--------------
008 TRIM15|Q9C019|276-465     --------------DGGGCTVGVAGEGVRRKGEMGL--------------
009 TRIM22|Q8IYM9|283-498     --------------GKIAWILGVHSKISSLNKRKSSGFAF----------
010 TRIM10|Q9UDY6|292-481     --------------HGGSCTVGVVSEDVQRKGELRL--------------
011 TRIM51|Q9BSJ1|269-452     --------------DSWNWAFGVCNNYWKEKRQNDK--------------
012 TRIM41|Q8WV44|413-630     --------------GRRGWAVGAARESTHHKEKVGPGGSSVGSGDASSSR
013 TRIM11|Q96F44|268-461     --------------DRTSWALGVCRENVNRKEKGEL--------------
014 TRIM7|Q9C029|324-511      --------------SKDGWAFGVARESVRRKGLTPF--------------
015 RNF39|Q9H2S5|210-420      ------SSGEDADDEESHYAVGAAGESVQRKGCVRL--------------
016 TRIM68|Q6AZZ1|285-481     --------------DRSEWGLGVCKQNVDRKEVVYL--------------
017 TRIM39|Q9HCM9|319-514     --------------DKTHWAVGVCRDSVSRKGELTP--------------
018 TRIM60|Q495X7|277-470     --------------NKPKWILGVCQDCLLRNWQDQP--------------
019 TRIM43|Q96BQ3|269-446     --------------NSCDWALGVCNNSWIRK--NST--------------
020 PYRIN|O15553|580-775      --------------DKTAWILGACKTSISRKGNMTL--------------
021 TRIM69|Q86WT6|305-500     --------------KKTKWTVGVVRESIIRKGSCPL--------------
022 ERMAP|Q96PL5|220-418      --------------DKTKWILGVCSESVSRKGKVTA--------------
023 TRIM75P|A6NK02|276-468    --------------DKSEWAIGICKDSLPTKARRPS--------------
024 TRIM35|Q9UPQ4|284-487     --------------GLQSWRVGVVRVRQDSGAEGHS--------------
025 TRIM77|I1YAP6|269-450     --------------DSCNWVIGLCREAWTKR--NDM--------------
026 TRIM9|Q9C026|533-702      -------------DNHPDPAFGVARMDVMKDVML----------------
027 TRIM65|Q6PJ69|313-506     ----------------HSVTLGVSYPQLPRCRLGPH-T------------
028 TRIM62|Q9BVG3|277-475     --------------EKTQWVIGLAHEAASRKGSIQI--------------
029 TRIM38|O00635|274-465     --------------EGTGWDLGVCMENVQRGTGMKQ--------------
030 BTN1A1|Q13410|285-479     --------------DRTDWAIGVCRENVMKKGFDPM--------------
031 BTN3A1|O00481|322-513     --------------DRKEWHIGVCSKNVQRKGWVKM--------------
032 TRIM25|Q14258|439-630     ----------------NFCGVGICYGSMNRQG--PE--------------
033 TRIM26|Q12899|295-539     LGDEEEEEEEEEEEVLESCMVGVARDSVKRKGDLSL--------------
034 TRIM58|Q8NG06|273-463     --------------EGAEWGLGVCQDTLPRKGETTP--------------
035 TRIM6|Q9C030|282-488      --------------KKTAWILGVCSNSLG------PTFSF----------
036 TRIM64|A6NGJ6|269-449     --------------LSSNWILGVCQDSRTA-DANFV--------------
037 TRIM21|P19474|268-465     --------------GKEAWDLGVCRDSVRRKGHFLL--------------
038 BTN2A1|Q7KYR7|310-506     --------------NVIEWTVGVCRDSVERKGEVLL--------------
039 TRIM67|Q6ZTA4|589-780     -------------DNHPDPAFGVARASVVKDMML----------------
040 TRIM34|Q9BYJ4|283-488     --------------KKTAWILGVYCRTYSRH----MKYVV----------
041 TRIM50|Q86XT4|276-475     --------------SKSDWRLGVIKGTASRKGKLNR--------------
042 TRIM4|Q9C037|288-500      --------------DSLEVAVGVCREDVMGITDRS---------------
```

```
001 TRIM14|Q14142|249-442     -----------ARLGCNRQSWCL-KRY-DLEYWAFHDGQ-----RSR-LR
002 TRIM47|Q96LD4|410-631     -----------GRLGRNAHSCCL-QWN-GRSFSVWFHGL-----EAP--L
003 TRIM49|P0CI25|269-452     -------------IDGKAGLFLLGCVKNDIQCSLFTTSP-----LML-QY
004 TRIM16|O95361|355-553     -----------SCISGNNFSWSL-QWN-GKEFTAWYSDM-----ETP--L
005 TRIM17|Q9Y577|277-475     --------------CPENGFWVV-QLSKGTKYLSTF-SA-----LTP-VM
006 TRIM27|P14373|298-492     --------------APQNGFWAV-SLWYGKEYWALT-SP-----MTA-LP
007 TRIM72|Q6ZMU5|271-475     --------------VPSQGLWLL-GLREGKILEAHVEAK----EPRALRS
008 TRIM15|Q9C019|276-465     --------------SAEDGVWAV-IIS-HQQCWAST-SP-----GTD-LP
009 TRIM22|Q8IYM9|283-498     -DPSVNYSKVYSRYRPQYGYWVI-GLQNTCEYNAFEDSSSSDPKVLT-LF
010 TRIM10|Q9UDY6|292-481     --------------RPEEGVWAV-RLA-WGFVSALG-SF-----PTR-LT
011 TRIM51|Q9BSJ1|269-452     -------------IDGEEGLFLLGCVKEDTHCSLFTTSP-----LVV-QY
012 TRIM41|Q8WV44|413-630     HHHRRRRLHLPQQPLLQREVWCV-GTN-GKRYQAQSSTE-----QTL-LS
013 TRIM11|Q96F44|268-461     --------------SAGNGFWIL-VFL-GSYY--NS-SE-----RAL-AP
014 TRIM7|Q9C029|324-511      --------------TPEEGVWAL-QLN-GGQYWAVT-SP-----ERS-PL
015 RNF39|Q9H2S5|210-420      --------------CPAGAVWAV-EGR-GGRLWALTAPE-----PTLLGG
016 TRIM68|Q6AZZ1|285-481     --------------SPHYGFWVI-RLRKGNEYRAGT-DE-----YPI-LS
017 TRIM39|Q9HCM9|319-514     --------------LPETGYWRV-RLWNGDKYAATT-TP-----FTP-LH
018 TRIM60|Q495X7|277-470     --------------SVLGGFWAI-GRYMKSGYVASG-PK-----TTQ-LL
019 TRIM43|Q96BQ3|269-446     -------------MVNSEDIFLLLCLKVDNHFNLLTTSP-----VFP-HY
020 PYRIN|O15553|580-775      --------------SPENGYWVV-IMMKENEYQASS-VP-----PTR-LL
021 TRIM69|Q86WT6|305-500     --------------TPEQGFWLL-RLRNQTDLKALD-LP-----SFS-LT
022 ERMAP|Q96PL5|220-418      --------------SPANGHWLL-RQSRGNEYEALT-SP-----QTS-FR
023 TRIM75P|A6NK02|276-468    --------------SAQQECWRI-E-LQDDGYHAPG-AF-----PTP-LL
024 TRIM35|Q9UPQ4|284-487     -------HS--CYHDTRSGFWYV-CRTQGVEGDHCV---TSDPATSP--L
025 TRIM77|I1YAP6|269-450     -------------RLDSEGIFLLLCLKVDDHFSLFSTSP-----LLP-HY
026 TRIM9|Q9C026|533-702      --------------GKDDKAWAM-YVDN-NRSWFMHNNS--HTNRTE--G
027 TRIM65|Q6PJ69|313-506     -----------DNIGRGPCSWGLCVQEDSLQAWHNGEAQ---------RL
028 TRIM62|Q9BVG3|277-475     --------------QPSRGFYCI-VMHDGNQYSACT-EP-----WTR-LN
029 TRIM38|O00635|274-465     --------------EPQSGFWTL-RLCKKKGYVALT-SP-----PTS-LH
030 BTN1A1|Q13410|285-479     --------------TPENGFWAV-ELY-GNGYWALT-PL-----RTP-LP
031 BTN3A1|O00481|322-513     --------------TPENGFWTM-GLTDGNKYRTLT-EP-----RTN-LK
032 TRIM25|Q14258|439-630     -----------SRLGRNSASWCV-EWF-NTKISAWHNNV-----EKT--L
033 TRIM26|Q12899|295-539     --------------RPEDGVWAL-RLS-SSGIWANT-SP-----EAE-LF
034 TRIM58|Q8NG06|273-463     --------------SPENGVWAL-WLLKGNEYMVLA-SP-----SVP-LL
```

```
035 TRIM6|Q9C030|282-488     -NHFAQNHSAYSRYQPQSGYWVI-GLQHNHEYRAYEDSSP----SLL-LS
036 TRIM64|A6NGJ6|269-449    -------------IDSDERFFLIS-SKRSNHYSLSTNSP-----PLI-QY
037 TRIM21|P19474|268-465    --------------SSKSGFWTI-WLWNKQKYEAGT-YP-----QTP-LH
038 BTN2A1|Q7KYR7|310-506    --------------IPQNGFWTL-EMHK-GQYRAVS-SP-----DRI-LP
039 TRIM67|Q6ZTA4|589-780    --------------GKDDKAWAM-YVDN-NRSWFMHCNS--HTNRTE--G
040 TRIM34|Q9BYJ4|283-488    -RRCANRQNLYTKYRPLFGYWVI-GLQNKCKYGVFEESLSSDPEVLT-LS
041 TRIM50|Q86XT4|276-475    --------------SPEHGVWLI-GLKEGRVYEAFACPR----VPL---P
042 TRIM4|Q9C037|288-500     ------------KMSPDVGIWAI-YWS-AAGYWPLIGFP-----GTP-TQ
```

```
001 TRIM14|Q14142|249-442    PRDDLDRLGVFLDYEAGVLAFYDVTGG--MSHLH----------------
002 TRIM47|Q96LD4|410-631    PHPFSPTVGVCLEYADRALAFYAVRD-GKMSLLRRLKASRPRRGGIPASP
003 TRIM49|P0CI25|269-452    IPKPTSRVGLFLDCEAKTVSFVDVNQ---SSLIY----------------
004 TRIM16|O95361|355-553    KAGPFRRLGVYIDFPGGILSFYGVEY-DTMTLVH----------------
005 TRIM17|Q9Y577|277-475    LMEPPSHMGIFLDFEAGEVSFYSVSD---GSHLH----------------
006 TRIM27|P14373|298-492    LRTPLQRVGIFLDYDAGEVSFYNVTE---RCHTF----------------
007 TRIM72|Q6ZMU5|271-475    PERRPTRIGLYLSFGDGVLSFYDASDADALVPLF----------------
008 TRIM15|Q9C019|276-465    LSEIPRGVRVALDYEAGQVTLHNAQT---QEPIF----------------
009 TRIM22|Q8IYM9|283-498    MAVPPCRIGVFLDYEAGIVSFFNVTNH--GALIY----------------
010 TRIM10|Q9UDY6|292-481    LKEQPRQVRVSLDYEVGWVTFTNAVT---REPIY----------------
011 TRIM51|Q9BSJ1|269-452    VPRPTSTVGLFLDCEGRTVSFVDVDQ---SSLIY----------------
012 TRIM41|Q8WV44|413-630    PSEKPRRFGVYLDYEAGRLGFYNAET---LAHVH----------------
013 TRIM11|Q96F44|268-461    LRDPPRRVGIFLDYEAGHLSFYSATD---GSLLF----------------
014 TRIM7|Q9C029|324-511     SCGHLSRVRVALDLEVGAVSFYAVED---MRHLY----------------
015 RNF39|Q9H2S5|210-420     VEPPPRRIRVDLDWERGRVAFYDGRS---LDLLY----------------
016 TRIM68|Q6AZZ1|285-481    LPVPPRRVGIFVDYEAHDISFYNVTDC--GSHIF----------------
017 TRIM39|Q9HCM9|319-514    IKVKPKRVGIFLDYEAGTLSFYNVTD---RSHIY----------------
018 TRIM60|Q495X7|277-470    PVVKPSKIGIFLDYELGDLSFYNMND---RSILY----------------
019 TRIM43|Q96BQ3|269-446    IEKPLGRVGVFLDFESGSVSFLNVTK---SSLIW----------------
020 PYRIN|O15553|580-775     IKEPPKRVGIFVDYRVGSISFYNVTA---RSHIY----------------
021 TRIM69|Q86WT6|305-500    LTNNLDKVGIYLDYEGGQLSFYNAKT---MTHIY----------------
022 ERMAP|Q96PL5|220-418     LKEPPRCVGIFLDYEAGVISFYNVTN---KSHIF----------------
023 TRIM75P|A6NK02|276-468   LEVKARAIGIFLDYEMGEISFYNMAE---KSHIC----------------
024 TRIM35|Q9UPQ4|284-487    VLAIPRRLRVELECEEGELSFYDAER---HCHLY----------------
025 TRIM77|I1YAP6|269-450    IPRPQGWLGVFLDYECGIVSFVNVAQ---SSLIC----------------
026 TRIM9|Q9C026|533-702     GITKGATIGVLLDLNRKNLTFFINDEQ--QGPIA----------------
027 TRIM65|Q6PJ69|313-506    PGVSGRLLGMDLDLASGCLTFYSLEPQ--TQPLY----------------
028 TRIM62|Q9BVG3|277-475    VRDKLDKVGVFLDYDQGLLIFYNADD---MSWLY----------------
029 TRIM38|O00635|274-465    LHEQPLLVGIFLDYEAGVVSFYNGNT---GCHIF----------------
030 BTN1A1|Q13410|285-479    LAGPPRRVGIFLDYESGDISFYNMND---GSDIY----------------
031 BTN3A1|O00481|322-513    LPKPPKKVGVFLDYETGDISFYNAVD---GSHIH----------------
032 TRIM25|Q14258|439-630    PSTKATRVGVLLNCDHGFVIFFAVAD-KVHLM-Y----------------
033 TRIM26|Q12899|295-539    PALRPRRVGIALDYEGGTVTFTNAES---QELIY----------------
034 TRIM58|Q8NG06|273-463    QLESPRCIGIFLDYEAGEISFYNVTD---GSYIY----------------
035 TRIM6|Q9C030|282-488     MTVPPRRVGVFLDYEAGTVSFYNVTNH--GFPIY----------------
036 TRIM64|A6NGJ6|269-449    VQRPLGQVGVFLDYDNGSVSFFDVSK---GSLIY----------------
037 TRIM21|P19474|268-465    LQVPPCQVGIFLDYEAGMVSFYNITDH--GSLIY----------------
038 BTN2A1|Q7KYR7|310-506    LKESLCRVGVFLDYEAGDVSFYNMRD---RSHIY----------------
039 TRIM67|Q6ZTA4|589-780    GVCKGATVGVLLDLNKHTLTFFINGQQ--QGPTA----------------
040 TRIM34|Q9BYJ4|283-488    MAVPPCRVGVFLDYEAGIVSFFNVTSH--GSLIY----------------
041 TRIM50|Q86XT4|276-475    VAGHPHRIGLYLHYEQGELTFFDADRPDDLRPLY----------------
042 TRIM4|Q9C037|288-500     QEPALHRVGVYLDRGTGNVSFYSAVD---GVH------------------
```

```
001 TRIM14|Q14142|249-442    ----------TFR--ATFQEPLYPALRLWEG---------AISIPRLP--
002 TRIM47|Q96LD4|410-631    IDPFQSRLDSHFA--GLFTHRLKPAFFLE---------SVDAHLQIGPLK
003 TRIM49|P0CI25|269-452    ----------TIPN-CSFSPPLRPIFCCIHF-------------------
004 TRIM16|O95361|355-553    ----------KFA--CKFSEPVYAAFWLS---------KKENAIRIVDLG
005 TRIM17|Q9Y577|277-475    ----------TYSQ-ATFPGPLQPFFCLGAPK---SGQMVISTVTMWV--
006 TRIM27|P14373|298-492    ----------TFSH-ATFCGPVRPYFSLS-Y----SGGKSAAPLIICPM-
007 TRIM72|Q6ZMU5|271-475    ----------AFH--ERLPRPVYPFFDVCWH----DKGKNAQPLLLVGPE
008 TRIM15|Q9C019|276-465    ----------TFT--ASFSGKVFPFFAVW---------KKGSCLTLKG--
009 TRIM22|Q8IYM9|283-498    ----------KFSG-CRFSRPAYPYFNPW---------NCLVPMTVCPPS
010 TRIM10|Q9UDY6|292-481    ----------TFT--ASFTRKVIPFFGLW---------GRGSSFSLSS--
011 TRIM51|Q9BSJ1|269-452    ----------TIPN-CSFSPPLRPIFCCSHF-------------------
012 TRIM41|Q8WV44|413-630    ----------TFSA-AFLGERVFPFFRVL---------SKGTRIKLCP--
013 TRIM11|Q96F44|268-461    ----------IFPE-IPFSGTLRPLFSPL--------SSSPTPMTICRPK
014 TRIM7|Q9C029|324-511     ----------TFR--VNFQERVFPLFSVC---------STGTYLRIWP--
015 RNF39|Q9H2S5|210-420     ----------AFQAPGPLGERIFPLFCTC---------DPRAPLRIVPAE
016 TRIM68|Q6AZZ1|285-481    ----------TFPR-YPFPGRLLPYFSPCYS----IGTNNTAPLAICSL-
```

```
017 TRIM39|Q9HCM9|319-514    -----------TFT--DTFTEKLWPLFYPGIR----AGRKNAAPLTIRPP-
018 TRIM60|Q495X7|277-470    -----------TFN--DCFTEAVWPYFYTG---------TDSEPLKICSVS
019 TRIM43|Q96BQ3|269-446    -----------SYPA-GSLTFPVRPFFYTGHR-------------------
020 PYRIN|O15553|580-775     -----------TFAS-CSFSGPLQPIFSPGTR----DGGKNTAPLTICPV-
021 TRIM69|Q86WT6|305-500    -----------TFS--NTFMEKLYPYFCPCLN----DGGENKEPLHILHPQ
022 ERMAP|Q96PL5|220-418     -----------TFT--HNFSGPLRPFFEPCLH----DGGKNTAPLVICSEL
023 TRIM75P|A6NK02|276-468   -----------TFT--DTFTGPLRPYFYVG---------PDSQPLRICTGT
024 TRIM35|Q9UPQ4|284-487    -----------TFH--ARF-GEVRPYFYLGGA----RGAGPPEPLRICPLH
025 TRIM77|I1YAP6|269-450    -----------SFLS-RIFYFPLRPFICHGSK-------------------
026 TRIM9|Q9C026|533-702     ------------FD---NVEGLFFPAVSLNRNVQVTLHTGLPVPDF-----
027 TRIM65|Q6PJ69|313-506    -----------TFH--ALFNQPLTPVFWLL----------EGRTLTLCHQP
028 TRIM62|Q9BVG3|277-475    -----------TFR--EKFPGKLCSYFSPGQSH---ANGKNVQPLRINTVR
029 TRIM38|O00635|274-465    -----------TFPK-ASFSDTLRPYFQVY----------QYSPLFLPPPG
030 BTN1A1|Q13410|285-479    -----------TFSN-VTFSGPLRPFFCLW--------SSGKKPLTICPIA
031 BTN3A1|O00481|322-513    -----------TFLD-VSFSEALYPVFRIL--------TLEPTALTICPA-
032 TRIM25|Q14258|439-630    -----------KFR--VDFTEALYPAFWVF---------SAGATLSICSPK
033 TRIM26|Q12899|295-539    -----------TFT--ATFTRRLVPFLWLK---------WPGTRLLLRP--
034 TRIM58|Q8NG06|273-463    -----------TFN--QLFSGLLRPYFFICD----------ATPLILPPTT
035 TRIM6|Q9C030|282-488     -----------TFSK-YYFPTTLCPYFNPC---------NCVIPMTLRRPS
036 TRIM64|A6NGJ6|269-449    -----------GFPP-SSFSSPLRPFFCFGCT-------------------
037 TRIM21|P19474|268-465    -----------SFSE-CAFTGPLRPFFSPGFN----DGGKNTAPLTLCPL-
038 BTN2A1|Q7KYR7|310-506    -----------TCPR-SAFSVPVRPFFRLG---------CEDSPIFICPAL
039 TRIM67|Q6ZTA4|589-780    ------------FS---HVDGVFMPALSLNRNVQVTLHTGLEVPTNLGRPK
040 TRIM34|Q9BYJ4|283-488    -----------KFSK-CCFSQPVYPYFNPW---------NCPAPMTLCPPS
041 TRIM50|Q86XT4|276-475    -----------TFQ--ADFQGKLYPILDTCWH----ERGSNSLPMVLPPPS
042 TRIM4|Q9C037|288-500     ---------------------------------------------------
```

```
001 TRIM14|Q14142|249-442    ----
002 TRIM47|Q96LD4|410-631    KSC-
003 TRIM49|P0CI25|269-452    ----
004 TRIM16|O95361|355-553    EEPE
005 TRIM17|Q9Y577|277-475    ----
006 TRIM27|P14373|298-492    ----
007 TRIM72|Q6ZMU5|271-475    GA--
008 TRIM15|Q9C019|276-465    ----
009 TRIM22|Q8IYM9|283-498    S---
010 TRIM10|Q9UDY6|292-481    ----
011 TRIM51|Q9BSJ1|269-452    ----
012 TRIM41|Q8WV44|413-630    ----
013 TRIM11|Q96F44|268-461    GGS-
014 TRIM7|Q9C029|324-511     ----
015 RNF39|Q9H2S5|210-420     S---
016 TRIM68|Q6AZZ1|285-481    ----
017 TRIM39|Q9HCM9|319-514    ----
018 TRIM60|Q495X7|277-470    DSE-
019 TRIM43|Q96BQ3|269-446    ----
020 PYRIN|O15553|580-775     ----
021 TRIM69|Q86WT6|305-500    ----
022 ERMAP|Q96PL5|220-418     HKS-
023 TRIM75P|A6NK02|276-468   VCE-
024 TRIM35|Q9UPQ4|284-487    ISV-
025 TRIM77|I1YAP6|269-450    ----
026 TRIM9|Q9C026|533-702     ----
027 TRIM65|Q6PJ69|313-506    GAV-
028 TRIM62|Q9BVG3|277-475    I---
029 TRIM38|O00635|274-465    D---
030 BTN1A1|Q13410|285-479    DGP-
031 BTN3A1|O00481|322-513    ----
032 TRIM25|Q14258|439-630    ----
033 TRIM26|Q12899|295-539    ----
034 TRIM58|Q8NG06|273-463    I---
035 TRIM6|Q9C030|282-488     S---
036 TRIM64|A6NGJ6|269-449    ----
037 TRIM21|P19474|268-465    ----
038 BTN2A1|Q7KYR7|310-506    TGA-
039 TRIM67|Q6ZTA4|589-780    L---
040 TRIM34|Q9BYJ4|283-488    S---
041 TRIM50|Q86XT4|276-475    G---
042 TRIM4|Q9C037|288-500     ----
```

1 2 3 4 5 6 7 8 9
Variable Average Conserved

X - Insufficient data - the calculation for this site was performed on less than 10% of the sequences.

Figure S4. Multiple sequence alignment of human B30.2 domains colored according to ConSurf conservation scores.

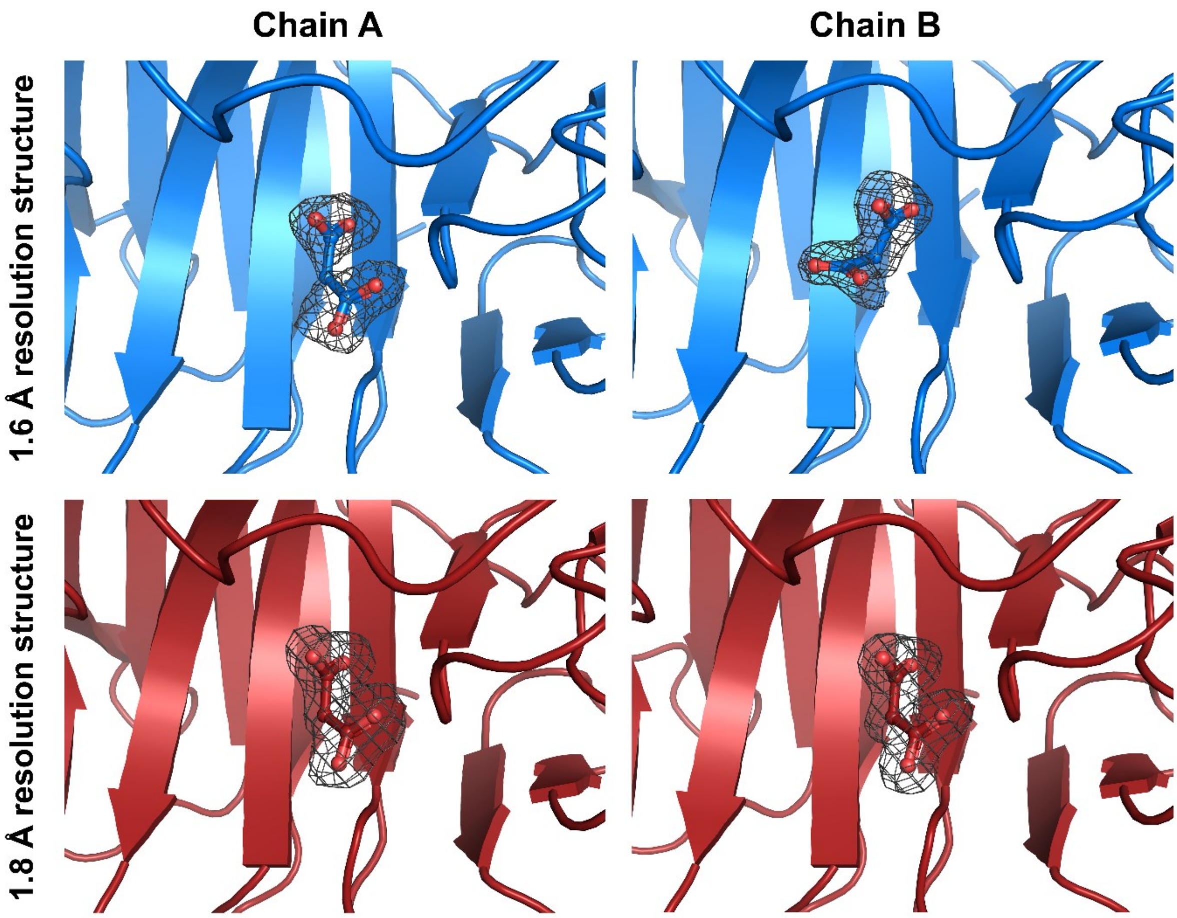

Figure S5. Malonate binding to TRIM7$^{B30.2}$. Omit electron density map contoured at the 2.5σ level for malonate bound to both chains of the asymmetric unit of the 1.6 Å (blue) and 1.8 Å (red) resolution structures.

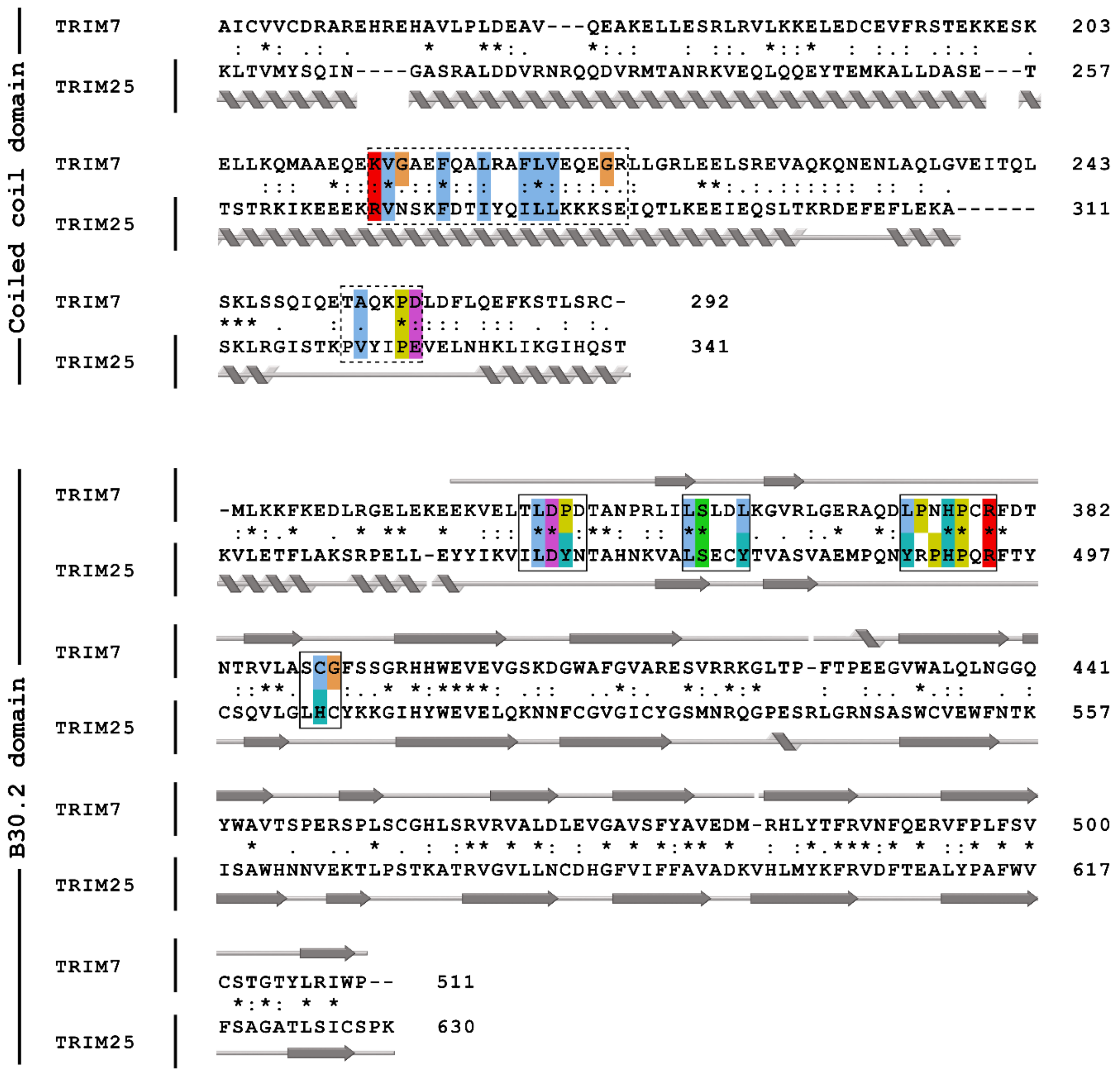

Figure S6. Sequence alignment of TRIM7 and TRIM25 coiled-coil and B30.2 domains. Extended version of Figure 5 including the complete sequence of both domains. Secondary structure elements observed in the crystal structures as assigned by DSSP (62) are indicated.

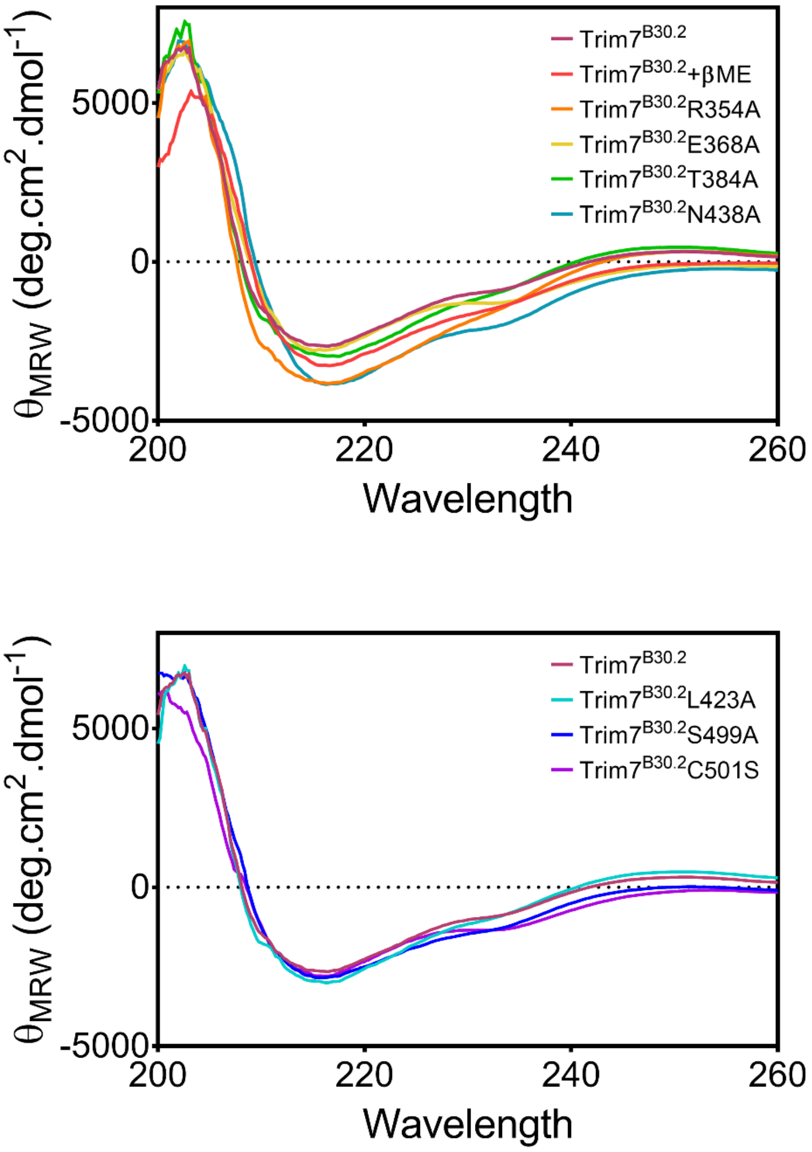

Figure S7. Far-UV CD spectra of TRIM7$^{B30.2}$ variants. The proteins were analyzed at a concentration of 10 µM. β-mercaptoethanol (βME) treated wild type TRIM7$^{B30.2}$ was purified and maintained in the presence of the reducing agent at 10 mM concentration which was reduced to 1.5 mM during the experiment.

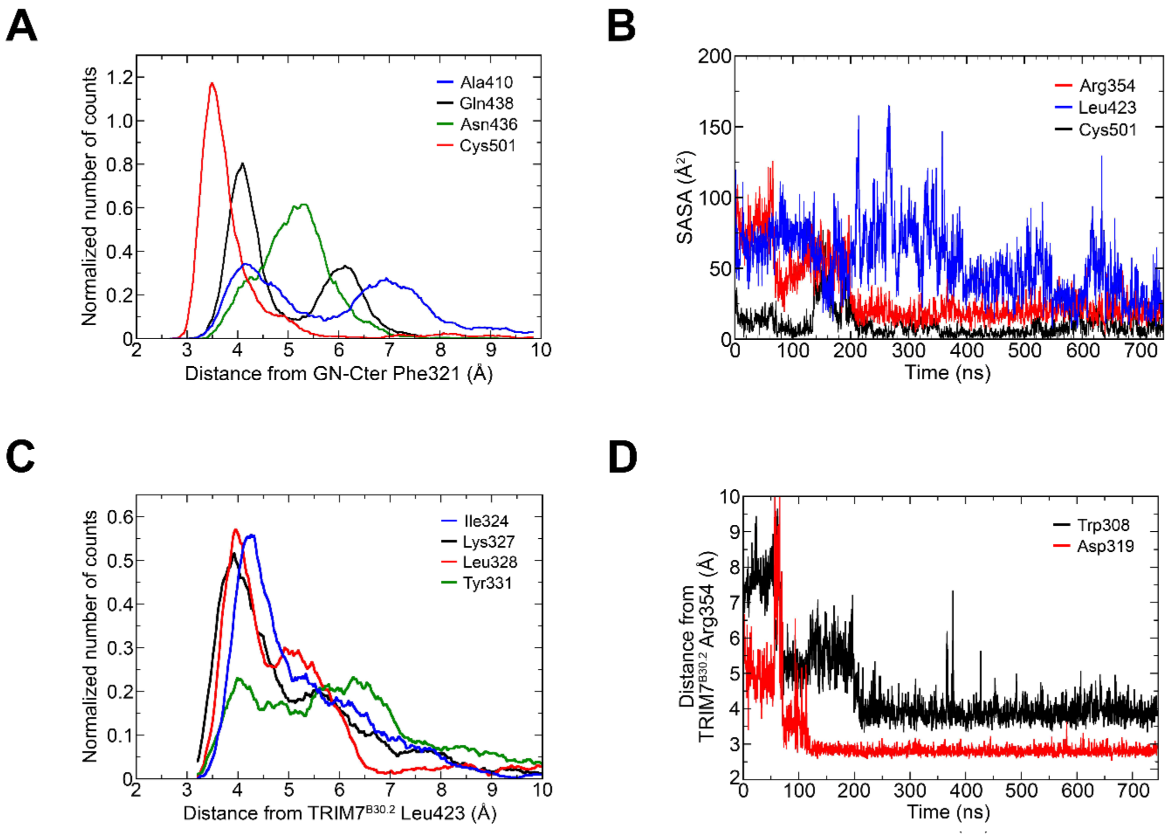

Figure S8. Analysis of interactions in the TRIM7$^{B30.2}$/GN1-Cter modeled complex during the MD simulation. (A) Histograms of distance between GN1-Cter Phe321 and Ala410, Asn436, Gln438 and Cys501 of TRIM7$^{B30.2}$. Cys501 is the closest contact, suggesting that it is the residue that will establish the strongest interaction. (B) Solvent accessible surface area (SASA) of Arg354, Leu423 and Cys501 as a function of simulation time. Cys501 is the less exposed residue, with a SASA value of ~35 Å at t=0 and ~5-10 Å at t=750 ns, while Leu423 goes from ~50 Å to ~20-25 Å, and Arg354 from to ~80 Å to ~20 Å. (C) Distance analysis for hydrophobic interactions of GN1-Cter amino acids (Ile324, Lys327, Leu328, Tyr331) with Leu423 of TRIM7$^{B30.2}$. Results are represented as normalized histograms. (D) Distance analysis during the simulation time for Arg354-Asp319 and Arg354-Trp308 interactions.

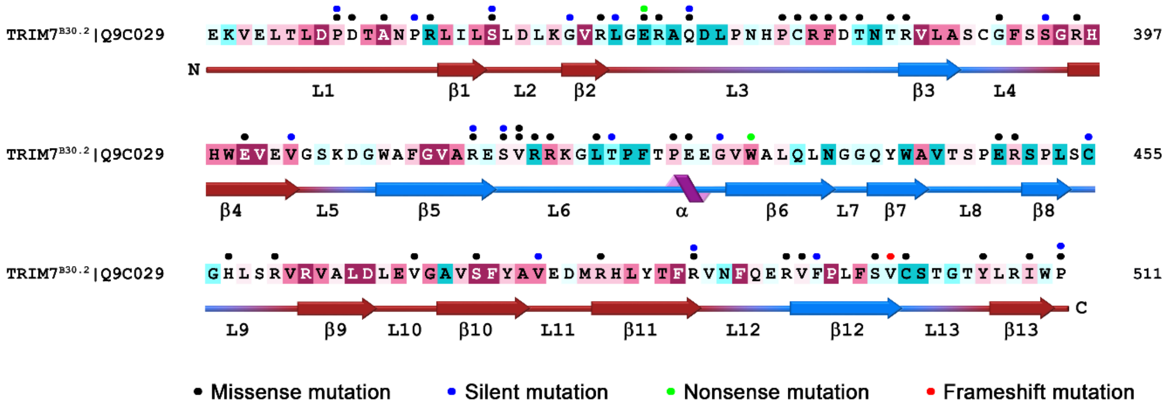

Figure S9. Cancer-associated somatic mutations identified in TRIM7$^{B30.2}$. Dots denote the location in the protein sequence of mutations referenced in the COSMIC (Catalogue Of Somatic Mutations In Cancer) database (44).

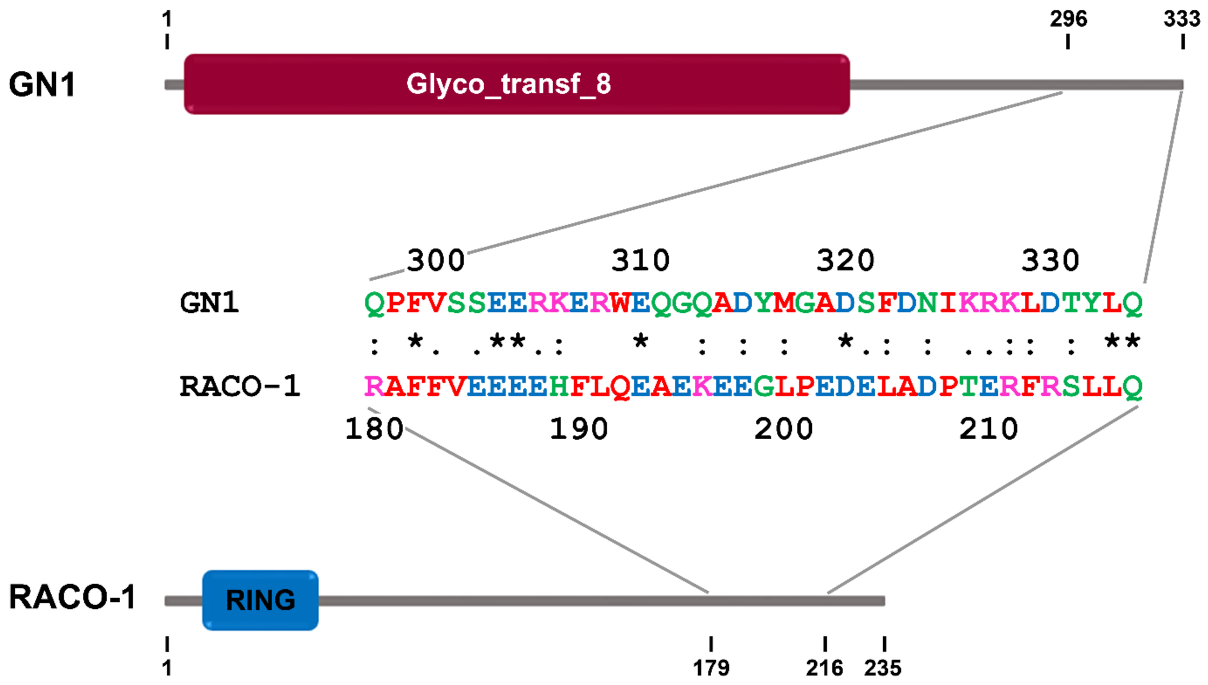

Figure S10. Sequence alignment of the C-terminal region of glycogenin-1 with RACO-1. RACO-1 region similar to glycogenin-1 C-terminus was identified by local sequence alignment using LALIGN (69).
